# Polyacrylic Acid‐Coated Selenium‐Doped Carbon Dots Inhibit Ferroptosis to Alleviate Chemotherapy‐Associated Acute Kidney Injury

**DOI:** 10.1002/advs.202400527

**Published:** 2024-04-30

**Authors:** Jiahuan Li, Chengcheng Fu, Baoli Feng, Qingquan Liu, Jiangjiang Gu, Mohammad Nauman Khan, Lvhui Sun, Honghong Wu, Hao Wu

**Affiliations:** ^1^ State Key Laboratory of Agricultural Microbiology College of Animal Science & Technology and College of Veterinary Medicine Huazhong Agricultural University Wuhan 430070 China; ^2^ Hubei Hongshan Laboratory Wuhan 430070 China; ^3^ MOA Key Laboratory of Crop Ecophysiology and Farming System in the Middle Reaches of the Yangtze River College of Plant Science & Technology Huazhong Agricultural University Wuhan 430070 China; ^4^ Shenzhen Institute of Nutrition and Health Huazhong Agricultural University Wuhan 430070 China; ^5^ Shenzhen Branch Guangdong Laboratory for Lingnan Modern Agriculture Genome Analysis Laboratory of the Ministry of Agriculture Agricultural Genomics Institute at Shenzhen Chinese Academy of Agricultural Sciences Shenzhen 518120 China; ^6^ Department of Nephrology Tongji Hospital Tongji Medical College Huazhong University of Science and Technology Wuhan 430030 China; ^7^ College of Chemistry Huazhong Agricultural University Wuhan 430070 China; ^8^ School of Breeding and Multiplication (Sanya Institute of Breeding and Multiplication) Hainan University Sanya 572000 China

**Keywords:** acute kidney injury, carbon dots, cisplatin, ferroptosis, selenium

## Abstract

Cisplatin‐associated acute kidney injury (AKI) is a severe clinical syndrome that significantly restricts the chemotherapeutic application of cisplatin in cancer patients. Ferroptosis, a newly characterized programmed cell death driven by the lethal accumulation of lipid peroxidation, is widely reported to be involved in the pathogenesis of cisplatin‐associated AKI. Targeted inhibition of ferroptosis holds great promise for developing novel therapeutics to alleviate AKI. Unfortunately, current ferroptosis inhibitors possess low bioavailability or perform non‐specific accumulation in the body, making them inefficient in alleviating cisplatin‐associated AKI or inadvertently reducing the anti‐tumor efficacy of cisplatin, thus not suitable for clinical application. In this study, a novel selenium nanomaterial, polyacrylic acid‐coated selenium‐doped carbon dots (SeCD), is rationally developed. SeCD exhibits high biocompatibility and specifically accumulates in the kidney. Administration of SeCD effectively scavenges broad‐spectrum reactive oxygen species and significantly facilitates GPX4 expression by releasing selenium, resulting in strong mitigation of ferroptosis in renal tubular epithelial cells and substantial alleviation of cisplatin‐associated AKI, without compromising the chemotherapeutic efficacy of cisplatin. This study highlights a novel and promising therapeutic approach for the clinical prevention of AKI in cancer patients undergoing cisplatin chemotherapy.

## Graphical abstract

Intravenously injected SeCD mainly accumulates in the kidney, where SeCD directly scavenges ROS and preserves mitochondrial integrity during the cisplatin challenge. Furthermore, SeCD releases selenium to facilitate GPX4 expression when encountering ROS under cisplatin exposure. Through these mechanisms, SeCD counteracts ferroptosis and alleviates cisplatin‐associated AKI, without compromising the anti‐tumor efficacy of cisplatin.

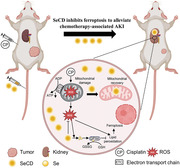

## 1 Introduction

Acute kidney injury (AKI) is a severe clinical syndrome with the incidence ranging from 10–15% in all hospitalizations.^[^ ^1^ ^]^ The sudden loss of glomerular filtration during AKI results in a drastic decrease in urine output and harmful retention of urea and creatinine in the serum.^[^ ^2^ ^]^ Cisplatin is a widely utilized chemotherapeutic drug for diverse human malignancies, including ovarian, breast, lung, and bladder cancers.^[^ ^3^ ^]^ However, its clinical application is hindered by the nephrotoxicity. Renal accumulation of cisplatin leads to ≈30% incidence of AKI in tumor patients receiving cisplatin chemotherapy.^[^ ^4^ ^]^ It is well‐established that cisplatin‐associated oxidative stress, DNA damage, and mitochondrial dysfunction would trigger renal tubular epithelial cell death and initiate renal inflammation.^[^ ^4^, ^5^ ^]^ Although some thiol‐producing compounds are effective in alleviating cisplatin‐induced nephropathy, their non‐specific accumulation in tumor tissues can inadvertently protect tumors and reduce the effectiveness of cisplatin chemotherapy.^[^ ^6^ ^]^ Therefore, there is a great urgency to develop novel strategies to alleviate cisplatin‐associated AKI without compromising the efficacy of cisplatin chemotherapy.

Ferroptosis is an iron‐dependent form of non‐apoptotic programmed cell death that was originally defined in 2012.^[^ ^7^ ^]^ The fundamental biochemical mechanism underlying ferroptosis is the deadly buildup of lipid peroxidation in phospholipids containing polyunsaturated fatty acids. This process is nonenzymatically driven by bioactive iron‐supported Fenton reaction and enzymatically catalyzed by lipoxygenases or cytochrome p450 oxidoreductase.^[^ ^7b^ ^]^ Lipid peroxidation disrupts bio‐membrane integrity and leads to an increase in bio‐membrane permeability, which eventually initiates the downstream death program, although the detailed mechanism by which lipid peroxidation drives ferroptosis has not been thoroughly understood. Conversely, several endogenous antioxidant systems have been identified to counteract ferroptosis. Specifically, the selenoprotein glutathione peroxidase 4 (GPX4) serves as the master ferroptosis suppressor and is capable of reducing lipid peroxides to generate corresponding lipid alcohols with the assistance of the co‐factor glutathione (GSH). Inhibiting GPX4 by RSL3^[^ ^8^ ^]^ or suppressing GSH biosynthesis by erastin (a typical ferroptosis inducer that binds to and inhibits system Xc^−^, thus restraining cystine uptake for GSH biosynthesis)^[^ ^7a^ ^]^ would potently boost lipid peroxidation and trigger ferroptosis.

It has been well understood that ferroptosis is implicated in diverse types of human diseases.^[^ ^7b^ ^]^ The pathological involvement of ferroptosis in kidney diseases has garnered significant attention. Ferroptosis synchronizes tubular cell death along entire tubule segments and aggravates inflammatory response‐related secondary tissue damages in the context of ischaemia/reperfusion‐induced AKI.^[^ ^9^ ^]^ Genetic ablation of GPX4 in mice results in acute renal failure. More importantly, the administration of a specific ferroptosis inhibitor effectively preserves renal physiological function and strongly mitigates tubular cell death.^[^ ^10^ ^]^ Additionally, cisplatin challenge leads to a notable cell death of proximal tubular epithelial cells, accompanied by the overwhelming accumulation of lipid peroxidation, GSH exhaustion, and GPX4 reduction, while this cell death and these typical ferroptosis characteristics are prominently diminished by the administration of a ferroptosis inhibitor.^[^ ^5^, ^11^ ^]^ Besides, ferroptosis is similarly engaged in the pathogenesis of other kinds of kidney injury, including septic AKI,^[^ ^12^ ^]^ folic acid‐induced AKI,^[^ ^12^ ^]^ diabetic nephropathy,^[^ ^13^ ^]^ and oxalate nephropathy.^[^ ^9^ ^]^ Therefore, the inhibition of ferroptosis holds great promise for the clinical treatment of AKI and nephropathy. Unfortunately, due to the low bioavailability or non‐specific accumulation in tumor tissues, current ferroptosis inhibitors are inefficient in alleviating AKI or would undesirably lower the anti‐tumor efficacy of cisplatin chemotherapy, thus not suitable for clinical application.^[^ ^14^ ^]^

Due to the inherently exceptional physicochemical properties, an increasing number of biocompatible nanomaterials have been intensively applied in biomedical fields, including drug delivery, clinical diagnosis, bio‐imaging, and disease therapy.^[^ ^15^ ^]^ Specifically, carbon dots are an emerging class of photoluminescent nanomaterials, characterized by a discrete quasi‐spherical structure with sizes less than 10 nm.^[^ ^16^ ^]^ Compared to small molecular drugs and other kinds of nanomaterials with bigger sizes, carbon dots can maximize renal retention for a considerably longer period.^[^ ^17^ ^]^ Furthermore, certain carbon dots exhibit reactive oxygen species (ROS) scavenging properties.^[^ ^18^ ^]^ Over the last decade, dozens of carbon dots have been synthesized and utilized to alleviate kidney injuries.^[^ ^19^ ^]^ Selenium is an essential trace element that is involved in a variety of fundamental physiological processes.^[^ ^20^ ^]^ Specifically, as a structural element for selenoproteins including GPXs and thioredoxin reductase, selenium is critical for the maintenance of intracellular redox homeostasis.^[^ ^21^ ^]^ Yao and colleagues presented compelling evidence to show that the selenium supplementation enhances GPX4 expression and mitigates ferroptosis, thus preserving helper T cells and promoting antibody responses in immunized mice and young adults with influenza vaccination.^[^ ^22^ ^]^ In addition, another elegant study reported that pharmacological selenium supplementation in the brain or systemic administration of a brain‐permeable selenopeptide facilitates GPX4 expression, and protects neurons from ferroptotic cell death, thus effectively improving behavior after hemorrhagic or ischemic stroke in mice.^[^ ^23^ ^]^ Nevertheless, excessive intake of inorganic or organic selenium compounds often results in extensive toxicity due to their narrow compatibility range. By contrast, selenium‐doped carbon dots present higher biocompatibility and are emerging in the treatment of diverse human diseases.^[^ ^24^ ^]^ However, the potential of biocompatible selenium‐doped carbon dots in mitigating ferroptosis and alleviating cisplatin‐associated AKI without compromising the efficacy of cisplatin chemotherapy remains unexplored.

Owing to the properties of renal retention, ROS scavenging, and potential anti‐ferroptosis of selenium‐doped carbon dots, a novel type of selenium‐doped carbon dots (SeCD) coated by polyacrylic acid, a polymer with extraordinary properties of excellent biocompatibility, biodegradability, and high degree of water dispersibility, was developed in this study. Intravenously injected SeCD exhibited good biocompatibility and was predominantly enriched in the kidneys of mice. More importantly, SeCD injection largely alleviated cisplatin‐associated AKI, without reducing the therapeutic efficacy of cisplatin in suppressing tumor growth in nude mice bearing xenograft tumors. Mechanistically, SeCD would release selenium to facilitate GPX4 expression, thereby enhancing the endogenous anti‐ferroptotic capacity. In addition, SeCD was capable to scavenge ROS directly and preserve mitochondrial integrity. By utilizing these mechanisms, SeCD counteracted ferroptosis in proximal tubular epithelial cells both in vivo and in vitro, thus mitigating cisplatin‐associated AKI. Overall, the excellent biocompatibility and robust anti‐ferroptotic capacity of SeCD make it a promising candidate for treating ferroptosis‐associated kidney diseases, especially in tumor patients who receive cisplatin chemotherapy.

## 2 Results

### 2.1 Preparation and Characterization of SeCD

**Figure 1:**
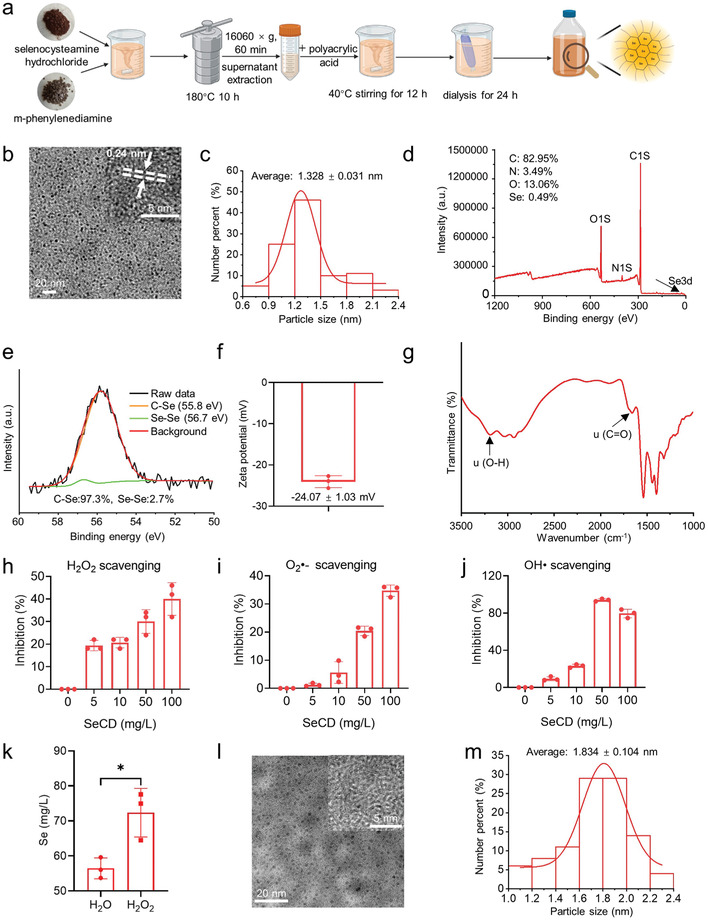
Synthesis and characterization of SeCD. a) Schematic illustration of SeCD synthesis (Produced by Biorender (www.biorender.com)). b) TEM image of SeCD. The inserted sub‐image showed a high‐resolution TEM image of SeCD. The TEM imaging was repeated three times independently with similar results. c) Collected particle size distribution ratio of 100 SeCD particles. d) XPS survey spectrum of SeCD. e) Se3d spectra in SeCD. f) Zeta potential of SeCD. g) FTIR spectra of SeCD. h–j) H_2_O_2_, O_2_^•‐^ and OH• scavenging activity of SeCD. k) Selenium release from SeCD in the presence of H_2_O or H_2_O_2_. l) TEM image of SeCD incubated in H_2_O_2_ solution. The inserted sub‐image showed a high‐resolution TEM image. m) Collected particle size distribution ratio of 100 SeCD particles incubated in H_2_O_2_ solution. For statistical analysis, data represent mean ± SD (*n* = 3). Statistical significance was calculated by using an unpaired two‐tailed Student's *t*‐test. *p < 0.05.

**Figure** **1**a illustrates the synthesis route of SeCD. Thermal processing and dialysis are the critical steps for SeCD synthesis. The morphology and size of SeCD were characterized by transmission electron microscope (TEM). TEM images showed that SeCD was well‐dispersed and exhibited a spherical morphology. The TEM size of SeCD was about 1.328 nm (Figure 1b,c). High‐resolution TEM imaging further revealed that the d‐space of SeCD was 0.24 nm (inset of Figure 1b), corresponding to the (002) lattice spacing between the graphene layers. The UV–vis spectra indicated that SeCD exhibited an absorption peak at 240 nm (Figure S1a, Supporting Information). Fluorescence spectra showed that SeCD had excitation‐dependent fluorescence properties (emission: 440–500 nm) under 400–700 nm excitation. The optimal excitation wavelength was about 440 nm, while the corresponding emission wavelength was about 510 nm (Figure S1b, Supporting Information). The X‐ray photoelectron spectroscopy (XPS) analysis confirmed that SeCD was mainly composed of carbon, nitrogen, oxygen, and selenium (Figure 1d,e and Figure S1c–e, Supporting Information), with the proportions of 82.95%, 3.49%, 13.06%, and 0.49%, respectively. The C1s spectrum displayed that SeCD had three obvious carbon structure peaks. These peaks were attributed to C─C/C═C (284.9 eV), C─O/C─N (285.5 eV), and C═O (288.9 eV) (Figure S1c, Supporting Information). In the N1s spectrum, the N─H bond at 399.8 eV and the C─N bond at 401.9 eV were observed (Figure S1d, Supporting Information). In the Se3d spectrum, the results showed that SeCD had C─Se bonds and Se─Se bonds at 55.8 and 56.7 eV, respectively. The ratio of the C─Se bonds and Se─Se bonds was 97.3% and 2.7%, respectively (Figure 1e). Zeta potential analysis showed that SeCD was negatively charged, with a value of −24.07 ± 1.03 mV (Figure 1f). Additionally, to investigate whether polyacrylic acid was successfully coated to SeCD, we characterized the sample with Fourier‐transform infrared spectroscopy (FTIR) analysis. Not surprisingly, a peak of the C═O group at 1651 cm^−1^ and a peak of the O─H group at 3196 cm^−1^ were shown in the SeCD sample (Figure 1g), indicating the successful coating of polyacrylic acid.

We further conducted in vitro experiments to assess the ROS scavenging abilities of SeCD. As depicted in Figure 1h–j, SeCD performed catalase‐like H_2_O_2_ scavenging activity, superoxide dismutase‐like O_2_•^−^ scavenging activity, as well as OH• scavenging capability. Generally, with the increase of SeCD concentration (0, 5, 10, 50, 100 mg L^−1^), the ROS scavenging abilities of SeCD were increased. At the concentration of 50 mg L^−1^, SeCD showed the strongest ability to scavenge OH• (Figure 1j). Furthermore, the ability of SeCD to release selenium in response to H_2_O_2_ was tested. Our results indicated that H_2_O_2_ treatment led to a markedly higher selenium release from SeCD when compared with SeCD incubated in H_2_O (72.35 versus 56.47 mg L^−1^ released selenium) (Figure 1k). This observation was further corroborated by TEM imaging, which showed that H_2_O_2_ exposure elevated the TEM size of SeCD to about 1.834 nm (Figure 1l,m), slightly bigger than the 1.328 nm TEM size of SeCD incubated in H_2_O (Figure 1b).

### 2.2 SeCD Releases Selenium and Protects Cells from Oxidative Damage

**Figure 2:**
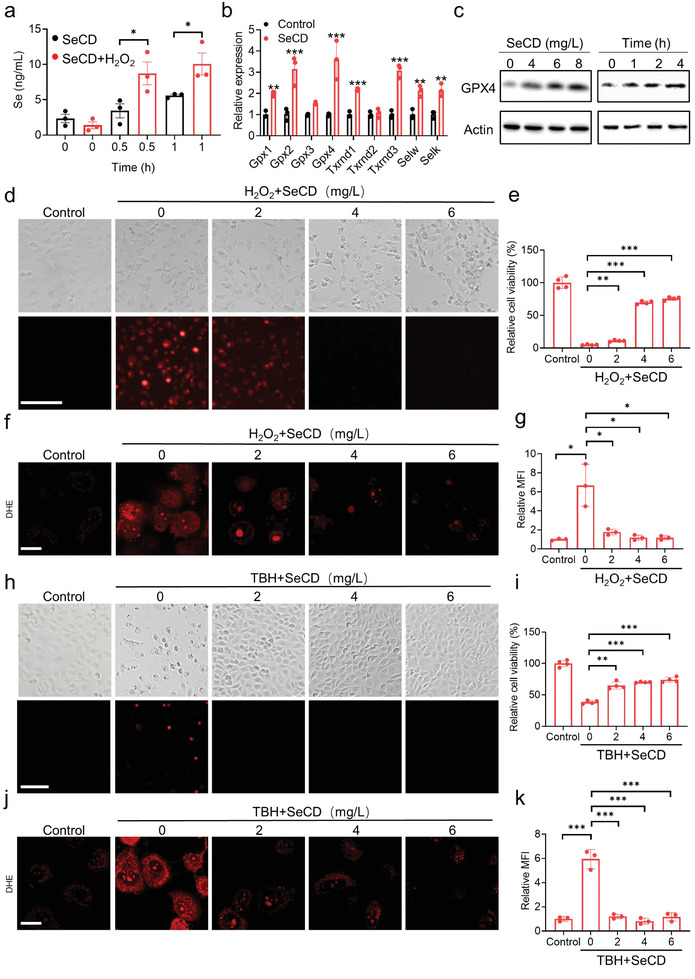
SeCD releases selenium and protects cells from oxidative damage. a) HK‐2 cells were incubated with 6 mg L^−1^ SeCD with or without 2 mM H_2_O_2_ for the indicated time. The released selenium in cells was determined by LC‐AFS. b) HK‐2 cells were treated with 6 mg L^−1^ SeCD for 4 h. Afterward, the selenoproteins‐encoded mRNAs were detected by quantitative real‐time PCR. c) HK‐2 cells were treated with the indicated dose of SeCD for 4 h, or treated with 6 mg L^−1^ SeCD for the indicated time, after which GPX4 expression was analyzed by Western blot. d,e) HK‐2 cells were treated with 2 mm H_2_O_2_ with or without the indicated dose of SeCD for 4 h. PI staining was used to visualize cell death (d). Scale bar: 100 µm. The relative cell viability was analyzed by CCK‐8 assay (e). f,g) HK‐2 cells were treated as in (d). Representative confocal microscopy images showed DHE staining (f). Scale bar: 20 µm. The relative MFI was calculated (g). h,i) HK‐2 cells were treated with 25 µm TBH with or without the indicated dose of SeCD for 4 h. PI staining was used to visualize cell death (h). Scale bar: 100 µm. The relative cell viability was analyzed by CCK‐8 assay (i). j,k) HK‐2 cells were treated as in (h). Representative confocal microscopy images showed DHE staining (j). Scale bar: 20 µm. The relative MFI was calculated (k). For statistical analysis, data represent mean ± SD (*n* = 3). Statistical significance was calculated by using an unpaired two‐tailed Student's *t*‐test. *p < 0.05, **p < 0.01, ***p < 0.001.

It should be noted that the cell medium and cellular compositions are more complicated than the in vitro test medium (which is H_2_O). Therefore, the other forms of ROS such as O_2_•^−^ and OH• might also accelerate the selenium release from SeCD in cells. To further investigate whether SeCD could release selenium in response to ROS in cultured cells, SeCD was incubated with human renal tubular epithelial cell HK‐2 in the presence or absence of H_2_O_2_. Liquid chromatography‐atomic fluorescence spectroscopy (LC‐AFS) analysis revealed that SeCD administration elevated intracellular selenium contents, while H_2_O_2_ presence facilitated this selenium release (**Figure** **2**a). Given that selenium is a structural element for selenoproteins, we subsequently examined the expressions of selenoproteins‐encoded genes upon SeCD administration. Quantitative real‐time PCR demonstrated that the majority of selenoproteins‐encoded genes were significantly increased, with the most pronounced upregulation being *Gpx4* (Figure 2b). Specifically, SeCD exposure increased the protein level of GPX4 in a dose‐ and time‐dependent manner (Figure 2c). These findings collectively suggested that SeCD releases selenium and expedites the expression of selenoproteins, especially GPX4.

The direct ROS scavenging activities and indirect antioxidant capacity by facilitating the expression of selenoproteins encouraged us to investigate the potential mitigation of oxidative damage by SeCD. We observed that SeCD exhibited an intensively inhibitory capacity on H_2_O_2_‐induced cell death in a dose‐dependent manner, as evidenced by propidium iodide (PI) staining and cell viability analysis (Figure 2d,e). Dihydroethidium (DHE) staining showed that supplementation of SeCD attenuated ROS generation upon H_2_O_2_ treatment (Figure 2f,g). Tert‐butyl hydroperoxide (TBH), an organic peroxide, is commonly utilized in oxidative stress studies.^[^ ^25^ ^]^ We observed a similar phenotype in TBH‐induced cell death. SeCD administration potently inhibited TBH‐induced cell death (Figure 2h,i) and mitigated ROS generation (Figure 2j,k) in HK‐2 cells.

### 2.3 Intravenously Injected SeCD Exhibits Good Biocompatibility and Mainly Enriches in the Kidney

**Figure 3:**
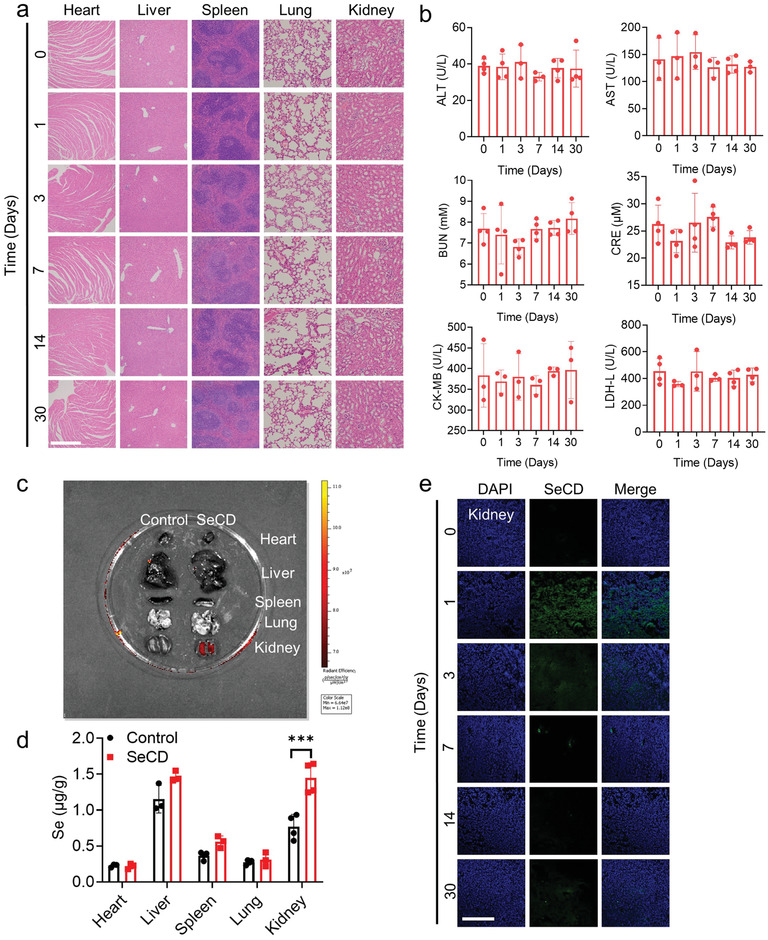
Intravenously injected SeCD exhibits good biocompatibility and mainly enriches in the kidneys in mice. a) H&E staining of indicated tissues isolated from mice after 1, 3, 7, 14, and 30 days of single SeCD intravenous injection (0.5 mg kg^−1^ bodyweight). Scale bar: 500 µm. b) Determination of serum ALT, AST, BUN, CRE, CK‐MB, and LDH‐L in mice after the indicated time of single SeCD intravenous injection. c) Representative ex vivo fluorescence imaging of tissues including the heart, liver, spleen, lung, and kidney isolated from mice after 1 day of intravenous SeCD administration (0.5 mg kg^−1^ bodyweight). d) Tissue selenium levels in the indicated tissues isolated from mice 1 day after SeCD injection. e) Representative fluorescence images of kidneys isolated from mice after 1, 3, 7, 14, and 30 days of SeCD injection (0.5 mg kg^−1^ bodyweight). The nuclei were stained with DAPI. Scale bar: 300 µm. For statistical analysis, data represent mean ± SD (*n* = 3–4). Statistical significance was calculated by using an unpaired two‐tailed Student's *t*‐test. ***p < 0.001.

To evaluate the biosafety of SeCD, SeCD was administered intravenously to mice at 0.5 mg kg^−1^ bodyweight. Tissues, including the heart, liver, spleen, lung, and kidney, were isolated 1, 3, 7, 14, or 30 days post‐injection. Histopathological examination showed no pathological changes in these tissues isolated from mice with SeCD administration, indicating good biosafety of SeCD (**Figure** **3**a). Additionally, serum biochemical analysis showed that SeCD injection did not change serum alanine aminotransferase (ALT) and serum aspartate aminotransferase (AST), two disease markers for liver failure, serum blood urea nitrogen (BUN) and serum creatinine (CRE), two disease markers for renal injury, as well as serum creatine kinase‐MB (CK‐MB) and serum lactate dehydrogenase‐L (LDH‐L), two disease markers of cardiac damage (Figure 3b). These findings suggest that intravenously injected SeCD is biocompatible in mice.

Fluorescence imaging revealed that intravenously injected SeCD was mainly enriched in the kidney, rather than the heart, liver, spleen, or lung (Figure 3c). This finding was subsequently validated by measurement of tissue selenium contents with LC‐AFS. SeCD injection led to a significant increase in selenium contents specifically in the kidney, but not in other tissues (Figure 3d). Selenium contents in the kidney were significantly increased 1 day post‐injection of SeCD, and then gradually decreased over time (Figure S2, Supporting Information). Subsequently, the predominant accumulation of SeCD in the kidney was further confirmed by tracking its fluorescence. The microscopy imaging results showed that SeCD fluorescence was indeed accumulated in the kidney shorter time post‐injection, then gradually diminished over time (Figure 3e).

### 2.4 SeCD Injection Alleviates Cisplatin‐Associated AKI and Mitigates Ferroptosis

**Figure 4:**
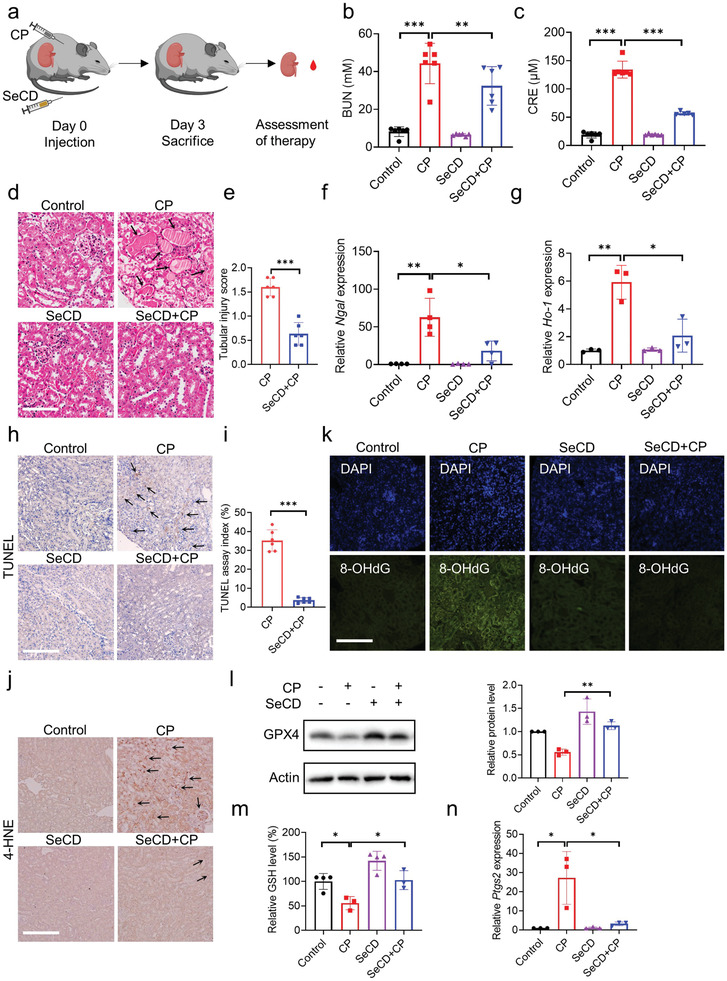
SeCD injection alleviates cisplatin‐induced AKI. a) The treatment schedule of cisplatin‐induced AKI and SeCD intervention in mice (Produced by Biorender (www.biorender.com)). b,c) Serum BUN (b) and CRE (c) in mice injected with vehicle, CP (20 mg kg^−1^ bodyweight), SeCD (0.5 mg kg^−1^ bodyweight), and CP plus SeCD, respectively. d) Representative H&E staining of the kidney tissues isolated from each group of mice. The black arrows indicate tubular necrosis, detachment of tubular epithelial cells, and cast formation. Scale bar: 200 µm. e) Tubular injury scores in H&E staining were calculated. f,g) Relative mRNA levels of *Ngal* (f) and *Ho‐1* (g) in the kidney tissues isolated from each group of mice. h) Representative TUNEL staining in the kidney tissues isolated from each group of mice. The black arrows show TUNEL‐positive staining. Scale bar: 200 µm. i) TUNEL assay indexes were calculated. j) Immunohistochemical staining of 4‐HNE in the kidney tissues. Positive staining is indicated by the black arrows. Scale bar: 200 µm. k) Immunofluorescence staining of 8‐OHdG in the kidney tissues. The nuclei were stained with DAPI. Scale bar: 200 µm. l) GPX4 expressions in the kidney tissues isolated from each group of mice. Relative protein levels were quantified and shown in the right histogram. m) Relative GSH levels in the kidney tissues isolated from each group of mice. n) Relative mRNA levels of *Ptgs2* in the kidney tissues isolated from each group of mice. For statistical analysis, data represent mean ± SD (*n* = 3–6). Statistical significance was calculated by using an unpaired two‐tailed Student's *t*‐test. *p < 0.05, **p < 0.01, ***p < 0.001.

Cisplatin is a widely used chemotherapeutic agent, but its application would initiate ferroptotic cell death in renal proximal tubules and trigger AKI.^[^ ^5^ ^]^ To investigate the potential protection of SeCD on cisplatin‐associated AKI, a cisplatin‐induced AKI mouse model was established and SeCD was administered intravenously for potential intervention (**Figure** **4**a). Serum biochemical analysis showed that the cisplatin challenge dramatically elevated BUN and CRE in serum, confirming the successful establishment of the cisplatin‐induced AKI mouse model (Figure 4b,c). Crucially, SeCD injection resulted in a significant reduction of serum BUN and CRE levels in cisplatin‐challenged mice (Figure 4b,c). The histopathological examination with hematoxylin & eosin (H&E) staining revealed extensive renal tubular damage in cisplatin‐treated mice, as manifested by tubular necrosis, tubular epithelial cell shed, and cast formation (Figure 4d,e). Notably, SeCD injection significantly ameliorated these pathological alterations (Figure 4d,e). Neutrophil gelatinase‐associated lipocalin (*Ngal*)^[^ ^26^ ^]^ and heme oxygenase‐1 (*Ho‐1*)^[^ ^27^ ^]^ serve as molecular markers for renal injury, and their expressions are substantially elevated during early AKI. Quantitative real‐time PCR analysis showed that cisplatin intoxication led to an obvious increase in the expressions of these two genes, whereas SeCD injection could substantially attenuate their expression levels (Figure 4f,g). Cisplatin‐associated AKI is accompanied by renal inflammation, as revealed by the increase in mRNA abundances of inflammatory cytokines *Tnf‐α* and *Il‐1β*, along with chemokines *Cxcl1*, *Cxcl2*, and *Ccl2*. The expressions of these mRNAs were significantly decreased by SeCD injection (Figure S3a–e, Supporting Information). Taken together, these findings suggested that intravenous SeCD injection could intensively ameliorate cisplatin‐associated AKI.

TdT‐mediated dUTP nick‐end labeling (TUNEL) analysis was performed to evaluate the potentiality of SeCD against cell death in vivo. The results demonstrated that SeCD significantly reduced the percentage of TUNEL‐positive cells in kidney tissues (Figure 4h,i), indicating that SeCD injection mitigated cisplatin‐induced cell death of tubular epithelial cells. This was further confirmed by an ex vivo experiment using isolated primary renal tubules. The long proximal tubule segments were quickly isolated for tissue culture. Cisplatin drove obvious cell death in these primary tubules, while this cell death could be substantially mitigated by SeCD administration, as evidenced by PI staining (Figure S4, Supporting Information).

Recently, it has been well characterized that ferroptosis is implicated in cisplatin‐associated AKI.^[^ ^28^ ^]^ We then investigated whether the protection of SeCD on the kidney under cisplatin challenge was attributable to the inhibition of ferroptosis. 4‐hydroxynonenal (4‐HNE) is a secondary aldehyde product of lipid peroxides and is widely utilized to characterize lipid peroxidation in vivo.^[^ ^29^ ^]^ Herein, we found that cisplatin treatment increased 4‐HNE in the kidney, which was significantly attenuated in the SeCD co‐treated group, as shown in the immunohistochemical staining by using a specific anti‐4‐HNE antibody (Figure 4j), Furthermore, lipid peroxides and the secondary products could attack DNA and initiate DNA oxidative damage, leading to the generation of 8‐hydroxydeoxyguanosine (8‐OHdG) adducts. Similarly, immunofluorescence staining employing an anti‐8‐OHdG antibody showed that SeCD injection robustly decreased 8‐OHdG signals (Figure 4k). Collectively, SeCD injection attenuated lipid peroxidation, the fundamental biochemical feature of ferroptosis. Moreover, SeCD injection mitigated GPX4 degradation and decelerated GSH exhaustion in the kidney (Figure 4l,m). The upregulation of *Ptgs2* mRNA has been regarded as a pharmacodynamic marker of ferroptotic cell death.^[^ ^30^ ^]^ We observed that cisplatin exposure resulted in increased *Ptgs2* expression, whereas SeCD injection reduced the abundance of *Ptgs2* mRNA (Figure 4n). Therefore, we concluded that intravenous SeCD injection prominently suppresses ferroptosis and alleviates cisplatin‐associated AKI.

### 2.5 SeCD Inhibits Ferroptosis in Cultured Renal Proximal Tubular Epithelium Cells

To further confirm the anti‐ferroptotic capacity of SeCD, HK‐2 cells were cultured for the following cytotoxicity analysis. First, fluorescence imaging and flow cytometry analysis suggested that SeCD entered cells in a time‐ and dose‐dependent manner (Figure S5a–f, Supporting Information). SeCD was internalized by the majority of cells within 15 min of exposure (Figure S5d–f, Supporting Information). In addition, the confocal fluorescence images suggested a punctate distribution of the intracellular SeCD (Figure S5a,d, Supporting Information).

**Figure 5:**
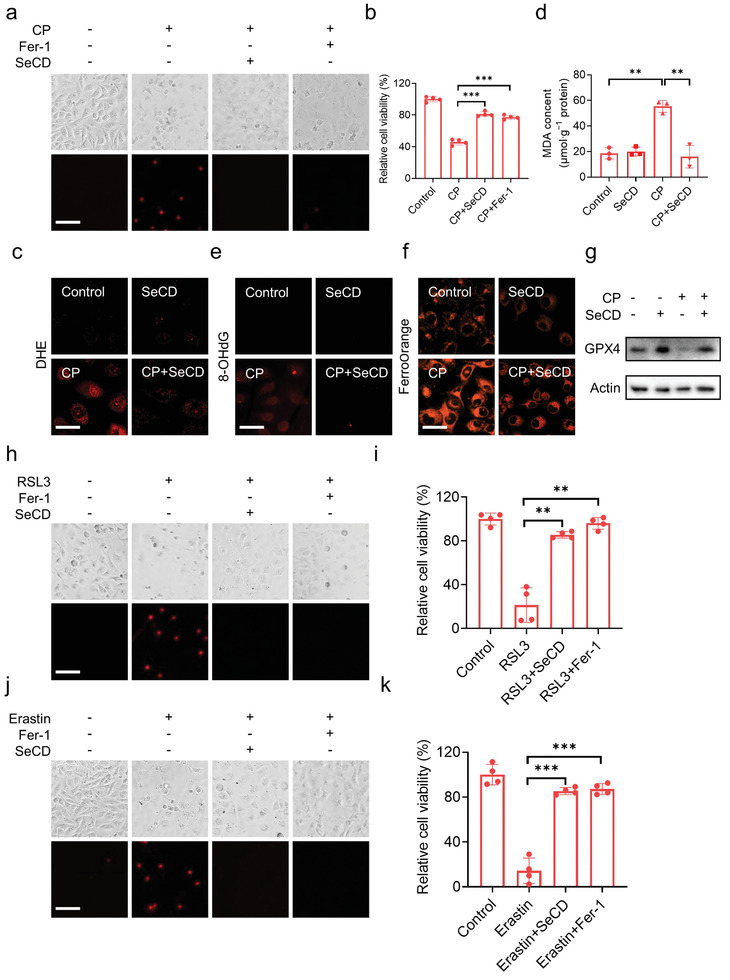
SeCD inhibits ferroptosis in cultured renal proximal tubular epithelium cells. a,b) HK‐2 cells were treated with 20 µm CP for 24 h in the presence or absence of 6 mg L^−1^ SeCD or 10 µm Fer‐1, then the cell death was visualized by PI staining (a). Scale bar: 100 µm. The relative cell viability was measured by CCK‐8 assay (b). (c) Confocal images showed DHE staining in HK‐2 cells with indicated treatment. Scale bar: 20 µm. (d) MDA contents in each group. e) Immunofluorescence staining of 8‐OHdG in HK‐2 cells with indicated treatment. Scale bar: 20 µm. f) Confocal images showed FerroOrange staining in HK‐2 cells with indicated treatment. Scale bar: 20 µm. g) HK‐2 cells were treated with 20 µM CP for 24 h in the presence or absence of 6 mg L^−1^ SeCD, and then GPX4 expression was detected by Western blot. h,i) HK‐2 cells were treated with 1 µM RSL3 for 4 h in the presence or absence of 6 mg L^−1^ SeCD or 10 µM Fer‐1, then the cell death was visualized by PI staining (h). Scale bar: 100 µm. The relative cell viability was measured by CCK‐8 assay (i). j,k) HK‐2 cells were treated with 10 µM erastin for 24 h in the presence or absence of 6 mg L^−1^ SeCD or 10 µM Fer‐1, then the cell death was visualized by PI staining (j). Scale bar: 100 µm. The relative cell viability was measured by CCK‐8 assay (k). For statistical analysis, data represent mean ± SD (*n* = 3–4). Statistical significance was calculated by using an unpaired two‐tailed Student's *t*‐test. ** *p* < 0.01, *** *p* < 0.001.

PI staining and microscopy imaging showed that cisplatin exposure triggered remarkable cell death in HK‐2 cells (**Figure** **5**a). This observation was further confirmed by cell viability measurement (Figure 5b). Administration of the specific ferroptosis inhibitor ferrostatin‐1 (Fer‐1) almost completely inhibited this cisplatin‐induced cell death (Figure 5a,b), indicating that cisplatin exposure initiated exact ferroptosis in HK‐2 cells. In this context, SeCD administration largely mitigated cisplatin‐induced ferroptosis, as evidenced by PI staining and cell viability analysis (Figure 5a,b). Moreover, SeCD provided prolonged protection (up to at least 72 h) of HK‐2 cells from cisplatin intoxication (Figure S6a, Supporting Information). Cisplatin expedited ROS generation in HK‐2 cells, as evidenced by DHE staining. Interestingly, cellular ROS was significantly reduced after SeCD treatment (Figure 5c). Additionally, cisplatin exposure led to an increase in cellular malonyl dialdehyde (MDA), a downstream product of lipid peroxidation (Figure 5d). SeCD administration dramatically reduced this massive MDA accumulation (Figure 5d). 8‐OHdG staining indicated that SeCD treatment significantly mitigated DNA oxidative damage induced by cisplatin (Figure 5e). Ferroptotic cells are characterized by the increase in intracellular free ferrous iron (Fe^2+^).^[^ ^31^ ^]^ By using FerroOrange, a commonly employed fluorescent probe labeling labile iron, confocal microscopy imaging showed that exposure to cisplatin elevated intracellular labile iron, whereas the fluorescence intensity of FerroOrange was significantly reduced in SeCD co‐treated cells, suggesting that SeCD decreased intracellular ferrous iron in cisplatin‐treated cells (Figure 5f). Furthermore, SeCD supplementation largely prevented GPX4 degradation during cisplatin exposure (Figure 5g). Collectively, these data suggested that SeCD mitigates cisplatin‐induced ferroptosis in HK‐2 cells.

We also investigated the potential protection of SeCD against ferroptosis induced by typical ferroptosis inducers, RSL3 and erastin. RSL3 covalently interacts with GPX4 to suppress its enzymatic activity of reducing lipid peroxidation.^[^ ^30a^ ^]^ Erastin binds to and inhibits system Xc‐, thereby restricting cystine uptake and decelerating GSH biosynthesis, leading to the inactivation of GPX4 indirectly.^[^ ^32^ ^]^ Notably, RSL3 and erastin treatment led to obvious cell death and a prominent decrease in cell viability of HK‐2 cells, while both SeCD and Fer‐1 supplementation potently prohibited this cell death (Figure 5h,j) and restored cell viability (Figure 5i,k). Moreover, SeCD exposure provided a long‐time protection. Microscopy imaging showed that RSL3 and erastin treatment failed to kill cells even up to 72 h when SeCD was supplemented (Figure S6b,c, Supporting Information). Similarly, we also observed that SeCD administration reduced MDA content (Figure S7a,b, Supporting Information), restrained ROS production (Figure S7c,d, Supporting Information), attenuated DNA oxidative damage (Figure S7e,f, Supporting Information), and decreased intracellular labile iron (Figure S7g,h, Supporting Information) in HK‐2 cells when challenged with RSL3 or erastin. In addition, co‐treatment with SeCD prevented GPX4 degradation in the presence of RSL3 or erastin (Figure S7i,j, Supporting Information). In summary, these findings demonstrated that SeCD is a powerful ferroptosis inhibitor and protects against ferroptosis triggered by diverse inducers.

### 2.6 SeCD Preserves Mitochondrial Integrity

**Figure 6:**
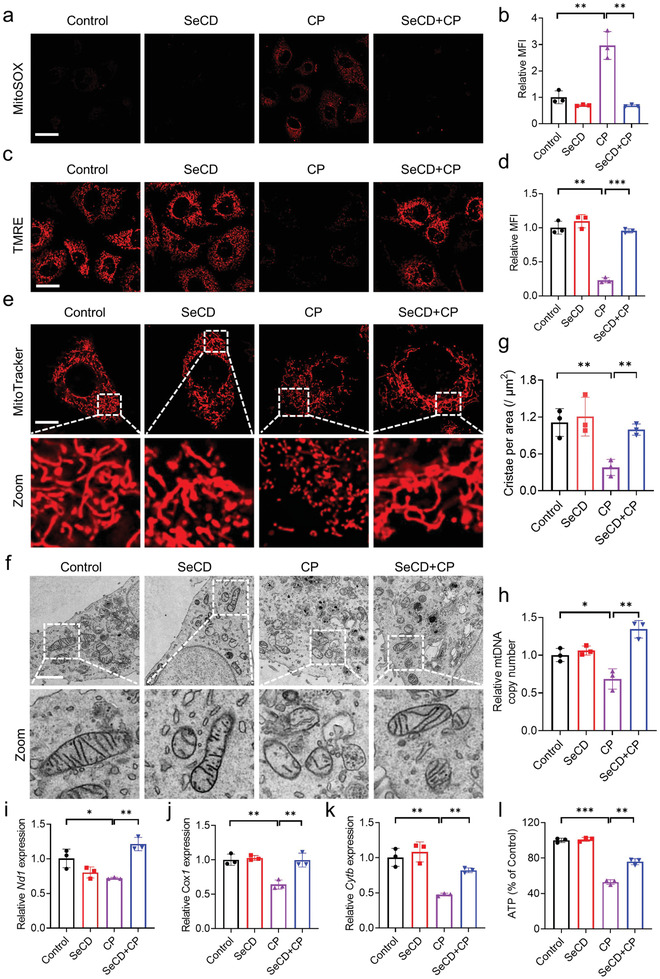
SeCD preserves mitochondrial integrity. a,b) HK‐2 cells were treated with 20 µM CP for 24 h with or without 6 mg L^−1^ SeCD. Representative confocal images were displayed to show MitoSOX staining (a). Scale bar: 20 µm. The relative MFI was calculated (b). c,d) Confocal images showed TMRE staining in HK‐2 cells with indicated treatment (c). Scale bar: 20 µm. The relative MFI was calculated (d). e) Confocal images showed MitoTracker staining in HK‐2 cells with indicated treatment. Scale bar: 20 µm. f,g) The Bio‐TEM images showed the mitochondrial morphology in HK‐2 cells with indicated treatment (f). Scale bar: 2 µm. The number of cristae per 1 µm^2^ mitochondrial area was calculated (g). h) Relative mtDNA copy number was determined by quantitative real‐time PCR in HK‐2 cells with indicated treatment. i–k) The expressions of mtDNA‐encoded mRNAs (*Nd1*, *Cox1*, and *Cytb*) were analyzed by quantitative real‐time PCR in HK‐2 cells with indicated treatment. l) The relative ATP contents in HK‐2 cells with indicated treatment were quantified. For statistical analysis, data represent mean ± SD (*n* = 3). Statistical significance was calculated by using an unpaired two‐tailed Student's *t*‐test. * *p* < 0.05, ** *p* < 0.01, *** *p* < 0.001.

It has been well characterized that cisplatin intoxication triggers mitochondrial damage,^[^ ^4^ ^]^ which is highly associated with ferroptosis vulnerability through multifaceted mechanisms.^[^ ^33^ ^]^ Confocal microscopy imaging followed by MitoSOX staining revealed that cisplatin intoxication resulted in excessive production of mitochondrial ROS, which was strongly decreased during SeCD supplementation (**Figure** **6**a,b). TMRE staining indicated that cisplatin led to mitochondrial depolarization, whereas SeCD treatment prominently preserved mitochondrial membrane potential (Figure 6c,d). In addition, we assessed mitochondrial morphology in HK‐2 cells by using MitoTracker staining. The result showed that most mitochondria exhibited long tubular morphology in a steady state. Cisplatin exposure led to mitochondrial fragmentation, whereas SeCD co‐treatment strongly preserved mitochondrial morphology (Figure 6e). Bio‐TEM imaging further verified that cisplatin exposure resulted in the abnormality of mitochondrial morphology. Specifically, cisplatin led to the decrease of mitochondrial cristae, which could be largely alleviated by SeCD administration (Figure 6f,g). Furthermore, we also observed that SeCD administration reduced mitochondrial ROS (Figure S7k,l, Supporting Information) and attenuated mitochondrial fission (Figure S7m,n, Supporting Information) when cells were exposed to RSL3 or erastin.

Early studies reported that cisplatin prefers to bind with mtDNA, thus leading to mitochondrial damage.^[^ ^34^ ^]^ Quantitative real‐time PCR suggested that the copy number of mtDNA was obviously decreased in cisplatin‐treated HK‐2 cells (Figure 6h), which is consistent with previous studies.^[^ ^35^ ^]^ In this context, cisplatin exposure resulted in an observable decrease in the abundance of mitochondrial mRNAs, including *Nd1*, *Cox1*, and *Cytb*, all of which are encoded by the mitochondrial genome (Figure 6i–k). Importantly, SeCD treatment mitigated this decrease in mtDNA copy number triggered by cisplatin exposure (Figure 6h), as well as preserved mitochondrial mRNAs (*Nd1*, *Cox1*, and *Cytb*) expression (Figure 6i–k). Mitochondria serve as the cellular powerhouse to produce ATP. SeCD administration effectively preserved mitochondrial ATP production during cisplatin exposure (Figure 6l). Taken together, these results indicated that SeCD effectively decreases mitochondrial oxidative damage and protects mitochondrial function upon pro‐ferroptotic insults. The preserved mitochondrial integrity may partially mitigate ferroptosis during the exposure of these ferroptosis inducers.^[^ ^36^ ^]^

The confocal fluorescence images suggested a punctate distribution of the intracellular SeCD (Figure S5a,d, Supporting Information). Next, we investigated whether the SeCD puncta had any mitochondrial localization or not. The MitoTracker‐SeCD imaging showed that a small part of the intracellular SeCD puncta co‐localized with MitoTracker (Figure S8, Supporting Information), preliminary indicating that SeCD may partially enrich in mitochondria, which possibly confers the mitochondrial protecting capacity of SeCD upon pro‐ferroptotic insults.

### 2.7 SeCD Improves Renal Health without Lowering the Chemotherapeutic Outcome of Cisplatin

**Figure 7:**
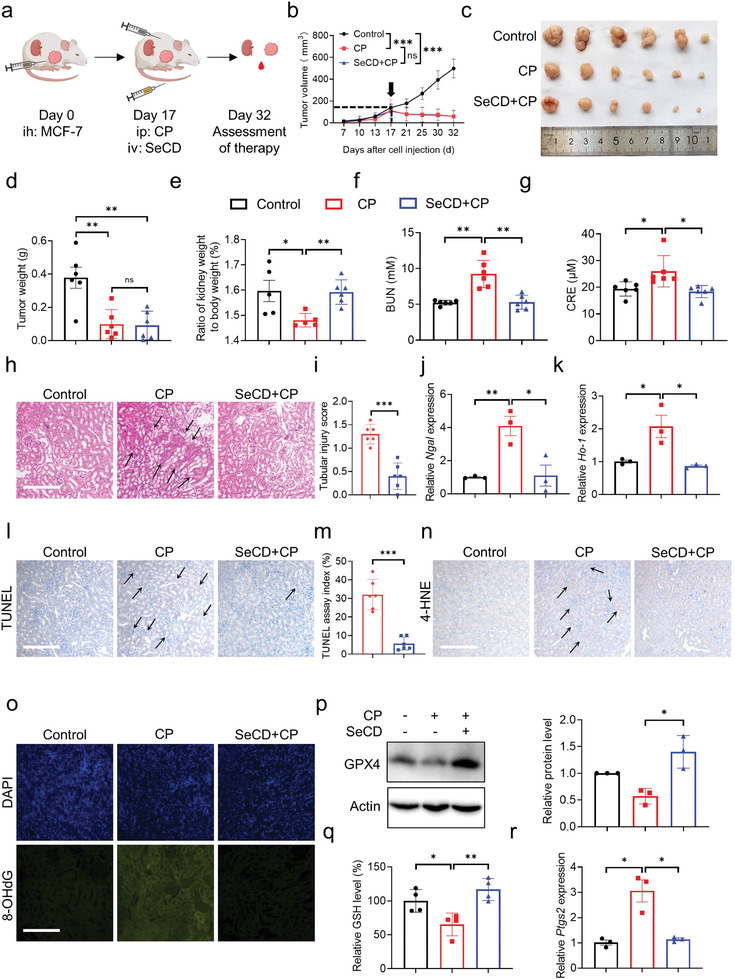
SeCD improves renal health without lowering the chemotherapeutic outcome of cisplatin. a) The treatment schedule of cisplatin chemotherapy and SeCD intervention in nude mice bearing MCF‐7 xenograft tumor. ih: hypodermic injection; ip: intraperitoneal injection; iv: intravenous injection (Produced by Biorender (www.biorender.com)). b) The tumor volume in each group. c) Images of the tumors in each group. d) The tumor weight in each group. e) The ratio of kidney weight to bodyweight in each group. f,g) Serum BUN (f) and CRE (g) in each group. h) Representative H&E staining of the kidney tissues in each group. The black arrows show sites of renal injury. Scale bar: 200 µm. i) Tubular injury scores in H&E staining were calculated. j,k) Relative mRNA levels of *Ngal* (j) and *Ho‐1* (k) in the kidney tissues isolated from each group of mice. l) Representative TUNEL staining in the kidney tissues isolated from each group of mice. The black arrows show TUNEL‐positive staining. Scale bar: 200 µm. m) TUNEL assay indexes were calculated. n) Immunohistochemical staining of 4‐HNE in the kidney tissues. Positive staining is indicated by the black arrows. Scale bar: 200 µm. o) Immunofluorescence staining of 8‐OHdG in the kidney tissues. The nuclei were stained with DAPI. Scale bar: 200 µm. p) GPX4 expressions in the kidney tissues isolated from each group of mice. Relative protein levels were quantified and shown in the right histogram. q) Relative GSH levels in the kidney tissues isolated from each group of mice. r) Relative mRNA levels of *Ptgs2* in the kidney tissues isolated from each group of mice. For statistical analysis, data represent mean ± SD (*n* = 3–6). Statistical significance was calculated by using two‐way ANOVA (b) or unpaired two‐tailed Student's *t*‐test (d–g, i–k, m, p–r). ns *p* > 0.05, * *p* < 0.05, ** *p* < 0.01, *** *p* < 0.001.

The favorable therapeutic efficacy of SeCD in alleviating cisplatin‐associated AKI by mitigating ferroptosis encouraged us to investigate whether SeCD could improve the renal health of tumor‐bearing mice that receive cisplatin chemotherapy. The xenograft tumor model was established in nude mice by subcutaneous injection of MCF‐7 human breast cancer cells. Following an optimal time for tumor growth, cisplatin was injected to suppress tumor growth, while SeCD was injected to potentially alleviate kidney damage (**Figure** **7**a). Promisingly, SeCD injection did not compromise the chemotherapeutic effect of cisplatin on the xenograft tumors, as evidenced by the equivalent tumor size and tumor weight in mice injected with cisplatin alone and injected with cisplatin plus SeCD (Figure 7b–d). H&E staining of the tumor tissues showed that cisplatin exposure induced extensive necrosis of tumor cells, while SeCD supplementation did not change the percentage of necrotic cells (Figure S9a, Supporting Information). This was confirmed by TUNEL staining. Cisplatin alone and cisplatin in combination with SeCD resulted in comparable necrosis of tumor cells (Figure S9b, Supporting Information). Ki‐67 is an important proliferation marker that has been widely used as a grading marker for many types of human cancers.^[^ ^37^ ^]^ Immunofluorescence staining showed that Ki‐67 positive cells were significantly reduced in both the cisplatin alone group and SeCD‐cisplatin co‐treatment group (Figure S9c, Supporting Information), further confirming that SeCD supplementation did not interfere with the chemotherapeutic efficacy of cisplatin. This is probably due to that SeCD failed to accumulate in the tumor tissues, as evidenced by the comparable selenium contents in tumor tissues isolated from three groups of mice (Figure S10, Supporting Information).

Next, we evaluated whether SeCD injection could still alleviate kidney injury in tumor‐bearing mice that receive cisplatin chemotherapy. SeCD administration increased the ratio of kidney weight to bodyweight, which was reduced in mice with cisplatin exposure (Figure 7e). In addition, we observed that cisplatin exposure resulted in a significant increase in serum BUN and CRE in tumor‐bearing mice, whereas SeCD administration significantly reduced serum BUN and CRE (Figure 7f,g). H&E staining further demonstrated that cisplatin‐induced tubular damage was significantly alleviated by SeCD injection (Figure 7h,i). Quantitative real‐time PCR showed that the expressions of *Ngal* and *Ho‐1* were elevated in the kidney tissues isolated from cisplatin‐treated tumor‐bearing mice, whereas dramatically decreased after SeCD treatment (Figure 7j,k).

To determine whether SeCD mitigated ferroptosis to alleviate renal injury in tumor‐bearing mice that receive cisplatin chemotherapy, we further examined ferroptotic characteristics in the kidney tissues. TUNEL staining demonstrated that SeCD injection significantly reduced cell death in the kidney tissues (Figure 7l,m). 4‐HNE‐positive staining and 8‐OHdG‐positive staining were significantly reduced after SeCD supplementation (Figure 7n,o). In addition, SeCD significantly upregulated the expression of GPX4 and decelerated the reduction of GSH (Figure 7p,q). We also examined the mRNA expression of the ferroptosis marker gene, *Ptgs2*, and found that cisplatin treatment induced a significant increase in *Ptgs2* mRNA, whereas SeCD administration reduced *Ptgs2* expression to the levels similar to those in the control group (Figure 7r). Taken together, these findings suggested that SeCD performs ideal protection against cisplatin‐induced renal injury in tumor‐bearing mice through mitigating ferroptosis, without lowering the chemotherapeutic outcome of cisplatin.

## 3 Discussion

Cisplatin‐associated AKI is a severe disease with high incidence and mortality, which largely limits the chemotherapeutic application of cisplatin. Recently, it has been widely reported that oxidative damage and ferroptosis in renal tubular epithelial cells play a pathological role in cisplatin‐associated AKI,^[^ ^28^ ^]^ raising an appealing insight that targeted inhibition of ferroptosis and scavenging ROS are promising for therapeutic design to treat cisplatin‐associated AKI in tumor patients. Traditional molecular antioxidants and nanomedicines with antioxidant capacities have been shown to mitigate oxidative damage in renal tubular epithelial cells, and thus effectively counteract ferroptotic programs to relieve cisplatin‐associated AKI.^[^ ^38^ ^]^ However, the non‐specific accumulation of these agents in tumor tissues would expedite tumor growth and facilitate therapeutic resistance.^[^ ^39^ ^]^ Herein, a novel type of nanoparticles, polyacrylic acid‐coated selenium‐doped carbon dots (SeCD), is rationally designed. SeCD is highly biocompatible and specifically accumulates in the kidneys of mice. SeCD can kill two birds with one stone. On the one hand, SeCD performs multiple antioxidant enzyme‐mimicking activities and directly scavenges ROS to alleviate oxidative damage. On the other hand, SeCD releases selenium when encountering ROS, facilitates GPX4 expression to strengthen the endogenous anti‐ferroptotic capacity. Administration of SeCD leads to strongly mitigate ferroptosis of renal tubular epithelial cells and effectively alleviate cisplatin‐associated AKI, without lowering the chemotherapeutic efficacy of cisplatin. Accordingly, the biocompatible SeCD is a promising drug candidate to treat AKI arising from diverse etiologies, particularly in tumor patients undergoing cisplatin chemotherapy.

Selenium‐doped carbon dots are an emerging hot topic in the biomedical field, due to the advantages of auto‐fluorescence properties, high biocompatibility, and potential ROS scavenging capabilities.^[^ ^40^ ^]^ For example, a previous study showed that selenium‐doped carbon dots can effectively remove free radicals and thus alleviate oxidative stress in subchondral bone for osteoarthritis treatment.^[^ ^24b^ ^]^ In this study, aiming to achieve both broad‐spectrum ROS scavenging and selenium release to activate the expression of selenoprotein GPX4 and counteract ferroptosis, we rationally designed ROS responsive SeCD with the ability to release selenium. The existence of C─Se and Se─Se groups in SeCD possibly accounts for the ROS responsiveness and selenium release properties. In addition, the mixing ratio of element valence state is known to be a base for ROS scavenging ability in nanomaterials.^[^ ^41^ ^]^

It has been well characterized that excessive ferroptosis in renal tubular epithelial cells is responsible for the pathogenesis of cisplatin‐associated AKI, and inhibition of ferroptosis through the administration of specific inhibitors is capable of alleviating this nephropathy.^[^ ^42^ ^]^ Oxidative damage‐associated lipid peroxidation and disturbed antioxidant systems constitute the fundamental biochemical features of ferroptosis.^[^ ^43^ ^]^ More importantly, the selenoprotein GPX4 is the sole antioxidant enzyme that could directly reduce lipid peroxidation to generate their corresponding lipid alcohols, thus being the master anti‐ferroptosis regulator in mammalian cells.^[^ ^30a^ ^]^ Administration of diverse types of selenium has been verified to expedite GPX4 expression and attenuate ferroptosis.^[^ ^22^ ^]^ Given the limited therapeutic window and narrow safety profile, traditional inorganic or organic selenium compounds are not suitable for disease treatment. In this study, our rationally designed SeCD would release selenium and facilitate GPX4 biosynthesis to repair lipid peroxidation when encountering ROS during the pathogenesis of AKI. More importantly, the small size of SeCD ensures a specific renal accumulation, but not any tumor enrichment, after intravenous injection in mice.^[^ ^44^ ^]^ This would avoid reducing the chemotherapeutic efficacy of cisplatin to suppress tumor growth, as scavenging ROS in cancer cells could spur the growth and metastasis of tumors.^[^ ^45^ ^]^

Mitochondria are the cellular powerhouse. Recent studies have underscored the pivotal role of mitochondria in ferroptosis regulation through multifaceted mechanisms.^[^ ^36^ ^]^ The mitochondrial tricarboxylic acid cycle and electron transport would facilitate mitochondrial ROS generation to sensitize ferroptosis.^[^ ^33^ ^]^ Our recent study suggested that mitochondrial redox homeostasis manipulated by the mitochondrial GSH pool could prevent ferroptosis and protect mice from doxorubicin‐induced cardiomyopathy.^[^ ^46^ ^]^ In addition, mtDNA release into the cytoplasm triggered by inhibition of DNA polymerase gamma, the DNA polymerase required for mtDNA replication,^[^ ^47^ ^]^ or mutation of Deoxyguanosine kinase, a rate‐limiting enzyme that manipulates precursor synthesis for mtDNA replication,^[^ ^48^ ^]^ would elevate ferroptosis vulnerability. In addition, mitochondrial damage has been widely reported in renal tubular epithelial cells upon cisplatin intoxication. Disturbed mitochondrial morphology, imbalanced mitochondrial redox homeostasis, and elevated mtDNA stress are pathologically manifested in cisplatin‐associated AKI.^[^ ^4^ ^]^ Specifically, mtDNA is particularly susceptible to cisplatin. Cisplatin exposure leads to mtDNA leakage and activation of the cGAS‐STING pathway, thereby aggravating renal inflammation and AKI progression.^[^ ^49^ ^]^ Cisplatin binding to mtDNA is also proposed to prevent mitochondrial DNA replication and transcription, resulting in an imbalance in mitochondrial proteomics and subsequent mitochondrial damage, as suggested by mitochondrial ROS burst, mitochondrial fragmentation, the reduction in mitochondrial cristae, mitochondrial depolarization, and reduced ATP production. SeCD administration potently preserves mitochondrial integrity in renal tubular epithelial cells under the exposure of cisplatin and typical ferroptosis inducers. This is probably due to that SeCD may partially enrich in mitochondria (Figure S8, Supporting Information).

## 4 Conclusions

In this study, we reported the biocompatible SeCD that could robustly alleviate cisplatin‐associated AKI by counteracting ferroptosis, without lowering the anti‐tumor efficacy of cisplatin. These desirable properties ensure that SeCD is a promising candidate to ameliorate the renal health of tumor patients receiving cisplatin chemotherapy.

## 5 Experimental Section

### Animal Experiments

8‐week‐old male C57BL/6J mice were purchased from the laboratory animal center of Huazhong Agricultural University, Wuhan, China. 6‐week‐old female BALB/c nude mice were purchased from Wuhan Moubaili Biotechnology, Wuhan, China. Mice were housed in a specific pathogen free animal facility at 24 °C with a 12‐h light/12‐h dark cycle and 40–60% humidity. Mice were given free access to water and food. The animal experiments were performed according to the procedures approved by the Scientific Ethics Committee of Huazhong Agricultural University (No. HZAUMO‐2023‐0229).

### Synthesis and Characterization of SeCD

SeCD was synthesized according to a previous study.^[^ ^50^ ^]^ Briefly, 100 mg selenocysteamine hydrochloride (99%, Sh‐yuan ye, Shanghai) and 50 mg m‐phenylenediamine (99.5%, McLean, Shanghai) were dissolved in 10 mL ultra‐pure water. The mixture was then transferred into a reactor which was injected with nitrogen to drive oxygen. The reactor was heated and stirred at 180 °C for 10 h, and the product was then centrifuged at 16 060× g for 60 min to collect the supernatant. Then, the supernatant was further mixed with a polyacrylic acid solution (2.5 g polyacrylic acid was dissolved in 5 mL ultra‐pure water). The mixture was stirred at 20× g for 24 h, then placed in a dialysis bag (molecular weight cut‐off = 3500 Da) for 24 h. The collected sample from the dialysis bag was the SeCD solution and stored in a refrigerator at 4 °C for further use. The concentration of SeCD was determined by inductively coupled plasma‐optical emission spectroscopy.

The Zeta potential of SeCD was determined by a 90 Plus PALS (Brookhaven Instruments, USA) as described in our previous study.^[^ ^51^ ^]^ 20 µL SeCD was titrated on a perforated carbon‐coated copper net and then imaged by a TEM (FEI Tecnai F20, Japan) with a 200 kV accelerating voltage. The fluorescence emission spectra of SeCD were analyzed by an F‐4600 FL spectrophotometer (Hitachi, Japan) in a quartz cuvette (10 × 10 mm). XPS analysis of SeCD was performed by using a K‐Alpha X‐ray photoelectron spectroscopy (Thermo Scientific, USA). The chemical characterization of SeCD by FTIR was performed by IS50 FTIR (Shimazu, Japan). The particle size of SeCD within TEM images was measured by using the Nano Measurer 1.2 software.

### In Vitro ROS Scavenging Activity Assay

In vitro ROS scavenging activity of SeCD was conducted according to our recent study.^[^ ^52^ ^]^ Briefly, H_2_O_2_ can react with molybdic acid to form a complex with a characteristic absorption peak at 405 nm. The content of H_2_O_2_ scavenged by SeCD was calculated by the absorbance decrease. The O_2_•^−^ scavenging activity assay was conducted by using a commercial kit (BC1415, Beijing Solarbio Science & Technology). For the OH• scavenging activity assay, the elimination of OH• by SeCD was detected by monitoring the absorbance of methyl violet (OH• can fade it from purple to a colorless appearance) at 582 nm.

### Cell Culture

Human kidney tubular epithelial HK‐2 cells and human breast cancer MCF‐7 cells were maintained in the lab. Cells were grown in DMEM (C11995500BT, Gibco) supplemented with 10% FBS (10099141C, Gibco) and 1% (v/v) penicillin/streptomycin (SV30010, HyClone) at 37 °C in a humidified atmosphere with 5% CO_2_.

### Cell Viability Assay and Cell Death Assay

For the cell viability assay, HK‐2 cells were seeded in 96‐well plates at 5000 cells well^−1^ for 24 h. Subsequently, the cells were subjected to indicated treatments as described in the corresponding experiments. The medium was discarded, and 100 µL CCK‐8 working solution was added into each well, followed by incubation at 37 °C for 1 h. The absorbance was quantified at 450 nm. Relative cell viability was calculated by normalizing each treatment to the control group. PI (HY‐D0815, MedChem Express) staining was utilized for cell death analysis. Cells were seeded at an appropriate density and cultured in normal conditions for 24 h. Afterward, the cells were treated with specific treatments as described in the corresponding experiments, then stained with PI working solution (5 µg mL^−1^ PI in PBS), after which images were captured by using a fluorescence microscope (IX73, Olympus, Japan).

### LC‐AFS analysis

To analyze selenium release following the administration of SeCD in vitro and in vivo, selenium levels were determined by LC‐AFS. For selenium release by SeCD in vitro, 6 mg L^−1^ SeCD was dissolved in water with or without H_2_O_2_. The solution was transferred directly to a dialysis bag. For selenium release by SeCD in vivo, HK‐2 cells were incubated with 6 mg L^−1^ SeCD for 0.5 or 1 h in the presence or absence of H_2_O_2_. Afterward, the cells were washed three times with PBS and harvested by trypsin digestion. Cells were counted and the same number of cells were lysed with 1 mL NP‐40, after which the cell lysate was transferred to a dialysis bag. The dialysis bags with the collected solution in vitro or cell lysate were placed in a 50 mL tube containing deionized water for 24 h at room temperature. At the end of dialysis, the solution in the 50 mL tube was collected and used for the subsequent LC‐AFS analysis. For LC‐AFS analysis, 1 mL obtained solution was transferred to a tube containing 10 mL HNO_3_. The samples were heated at 180 °C for 1.5 h, then the tube was uncovered and heated until the liquid had completely evaporated. 4 mL HCl and 4 mL H_2_O were added to the tube. The tube was heated at 110 °C for another 1.5 h, then uncovered and heated until 2 mL liquid remained. 10% HCl was added to the tube to dissolve the liquid. The solution was then transferred to a 50 mL centrifuge tube, 8% potassium ferrocyanide was added and the mixture was allowed to stand for 1 h. Finally, the mixture's volume was adjusted to 25 mL by dilution with water. The selenium standard solutions were prepared similarly to produce five of the standard solutions of gradient concentrations. The selenium contents were determined by a liquid chromatography‐atomic fluorescence coupler (LC‐AFS8530, Thermo Fisher Scientific, America).

### Biosafety Evaluation of SeCD

8‐week‐old male C57BL/6J mice were intravenously injected with SeCD at 0.5 mg kg^−1^ bodyweight. At 1, 3, 7, 14, and 30 days post‐injection, mice were sacrificed, and blood samples were collected. Serum was obtained by centrifugation of the blood at 860× g for 10 min at 4 °C. Serum ALT, AST, BUN, CRE, CK‐MB, and LDH‐L were measured by using an automatic biochemistry analyzer. Meanwhile, tissues, including the heart, liver, spleen, lung, and kidney, were obtained and sectioned for H&E staining. A microscope (BX53, Olympus, Japan) was employed to observe the pathological changes in these tissues.

### Biodistribution Analysis of SeCD

To assess the biodistribution of SeCD in mice, C57BL/6J mice were intravenously injected with SeCD at 0.5 mg kg^−1^ bodyweight. Tissues, including the heart, liver, spleen, lung, and kidney, were isolated 1, 3, 7, 14, and 30 days post‐injection. These tissues were embedded in OCT (G6059, Servicebio), frozen in liquid nitrogen for 5 min, and sectioned to a thickness of 10 µm. After being washed three times with PBS, the tissue sections were sealed with antifade mounting medium supplemented with DAPI (P0131, Beyotime Biotechnology), and images were captured to analyze the SeCD fluorescence distributing in these tissues by using a microscope (N‐STORM, Nikon, Japan). In addition, the selenium contents in these tissues were determined by LC‐AFS analysis to further analyze the biodistribution of SeCD in mice.

### Fluorescence Imaging of SeCD in Isolated Tissues

8‐week‐old male C57BL/6J mice were intravenously injected with SeCD at 0.5 mg kg^−1^ bodyweight. Mice were sacrificed 24 h post‐injection. Tissues, including the heart, liver, spleen, lung, and kidney, were isolated immediately. The distribution of SeCD within these tissues was visualized with an IVIS spectral imaging system (PerkinElmer, USA).

### Establishment of the Mouse AKI Model and SeCD Intervention

8‐week‐old male C57BL/6J mice were received a single intraperitoneal injection of cisplatin (dissolved in warm saline at 1 mg mL^−1^) at 20 mg kg^−1^ bodyweight. Simultaneously, SeCD was intravenously injected at 0.5 mg kg^−1^ bodyweight for intervention. Control animals were injected with the same volume of sterilized saline. After injection, the mice were given free access to water and food, and sacrificed 72 h post‐injection. Blood and kidneys were collected for the following analysis.

### H&E Staining

The fresh tissues were fixed in 4% paraformaldehyde, embedded in paraffin, and sectioned to a thickness of 10 µm. Following dewaxing and hydration, sections were stained as follows: hematoxylin staining for 10 min, differentiation in 1% hydrochloric acid‐ethanol solution for 5 s, incubation in tap water for 10–20 min, eosin staining for 2 min and rinsed in pure water. After dehydration, sections were embedded in neutral resin. Images were taken under a microscope (BX53, Olympus, Japan) to assess the histopathological changes, and five views were randomly photographed for each section. The histopathological changes were scored semi‐quantitatively in a double‐blind fashion on a scale of 0 to 4 according to the percentage of damaged tubules (0, no damage; 1, <25% damage; 2, 25–50% damage; 3, 50–75% damage; 4, >75% damage).

### TUNEL Staining

Tissues were fixed in 4% paraformaldehyde, embedded in paraffin, and serially sectioned. TUNEL staining was performed by using a TUNEL assay kit (C1098, Beyotime Biotechnology) according to the manufacturer's instructions. Images were taken under a microscope (BX53, Olympus, Japan), and five views were randomly photographed for each section. TUNEL‐positive cells were counted in each section in a double‐blind manner, and the TUNEL assay index was expressed as the percentage of TUNEL‐positive cells in the sections.

### 4‐HNE Staining

Paraffin sections were treated with 3% aqueous H_2_O_2_ for 10 min at room temperature after dewaxing and hydration. The sections were boiled in citrate buffer (10 mM sodium citrate, pH 6.0) for 10 min for antigen retrieval. The sections were then incubated with the anti‐4‐HNE antibody (ab46545, Abcam) at 4 °C overnight and the secondary antibody at 37 °C for 30 min, successively. DAB substrate solution (G1212, Servicebio) was added to reveal the color of antibody staining. The nuclei were re‐stained with hematoxylin (G1004, Servicebio). The sections were sealed in neutral resin before being observed under a microscope. For 4‐HNE staining, 6 mice per group were used.

### 8‐OHdG Staining

After dewaxing and hydration, tissue sections were boiled in citrate buffer for 15 min. Afterward, sections were permeabilized using 0.1% Triton X‐100 for 10 min. After 1 h of block with 5% goat serum, the sections were incubated with the anti‐8‐OHdG antibody (SC‐66036, Santa Cruz Biotechnology) at 4 °C overnight, and the anti‐mouse IgG Alexa Fluor 488 (A11001, Thermo Fisher Scientific) secondary antibody at room temperature for 1 h in the dark. Next, the sections were sealed with an antifade mounting medium supplemented with DAPI. Images were captured by using a fluorescence microscope (BX53, Olympus, Japan). For 8‐OHdG staining, 6 mice per group were used.

### GSH Measurement

20 mg fresh kidney tissues were used for GSH measurement by using a commercially available kit (S0052, Beyotime Biotechnology) according to the manufacturer's instructions.

### MDA Measurement

HK‐2 cells and fresh kidney tissues were homogenized with RIPA lysis buffer (P0013B, Beyotime Biotechnology), and then the homogenate was centrifuged at 10 000× g for 10 min at 4 °C. The supernatant obtained was used to determine MDA contents by using a commercially available kit (S0131S, Beyotime Biotechnology) according to the manufacturer's instructions. In addition, the protein concentration was determined by using a BCA kit (P0011, Beyotime Biotechnology), and the relative MDA levels were normalized to the protein contents.

### RNA Extraction and Quantitative Real‐Time PCR

Total RNA was extracted from tissues or cultured cells by using a Trizol reagent (RK30129, ABclonal). RNA concentration was quantified by using a NanoDrop 2000 instrument (Thermo Fisher Scientific). 1 µg RNA was reversely transcribed into cDNA by using ABScript III RT Master Mix (RK20429, ABclonal) and subjected to quantitative real‐time PCR with 2 × Universal SYBR Green Fast qPCR Mix (RK21203, ABclonal). The quantitative real‐time PCR was performed in a total reaction volume of 10 µL, consisting of 5 µL 2 × Universal SYBR Green Fast qPCR Mix, 2 µL ddH_2_O, 0.5 µL of each primer (10 µm), and 2 µL diluted cDNA template. The reaction was initiated at 95 °C for 3 min, followed by 40 cycles: 95 °C for 5 s, 60 °C for 30 s, 72 °C for 20 s. Relative mRNA levels were estimated by using the 2^−∆∆CT^ method and normalized to *Actin*. The sequences of the primers used in this study are listed in Table S1, Supporting Information.

### Protein Preparation and Western Blot

Mouse tissues or cultured cells were lysed with RIPA buffer (P0013B, Beyotime Biotechnology) supplemented with protease inhibitors on ice for 30 min. The lysate was centrifuged at 10 000× g for 10 min at 4 °C. The supernatant was collected and the protein concentration was analyzed by using a BCA kit (P0011, Beyotime Biotechnology). Protein samples were separated in SDS‐PAGE (8–12%) and then transferred onto PVDF membranes. The membranes were immersed in 5% milk in TBST buffer (formulated as 137 mm NaCl, 20 mm Tris, 0.1% Tween‐20, pH 7.6) for 1 h at room temperature, followed by incubation with primary antibodies against Actin (AC026, ABclonal, 1:10 000 dilution) or GPX4 (ab125066, Abcam, 1:1000 dilution) at 4 °C overnight. The membranes were then washed three times with TBST buffer and incubated with the secondary antibody (7074S, Cell Signaling Technology, 1:10 000 dilution) for 1 h at room temperature. The protein bands were visualized by adding chemiluminescence reagents.

### Isolation of Primary Renal Tubules

After the mice were sacrificed, the kidneys were promptly isolated. The renal capsule and medulla were first removed. The remaining tissues were minced into small pieces in Krebs‐Ringer buffer (PB180347, Procell Life Science & Technology), followed by digestion in 1 mg mL^−1^ type I collagenase (A004194‐0100, Sangon Biotech) at 37 °C for 10 min. Post‐digestion, undigested tissues were discarded by using a cell strainer with 224 µm pores (F513450‐0001, Sangon Biotech), followed by filtration through another cell strainer with 100 µm pores (F613463‐0001, Sangon Biotech). The long proximal tubule segments remaining in the cell strainer with 100 µm pores were collected and transferred to a 24‐well plate for subsequent culture, with 500 µL medium per well.

### Flow Cytometry Analysis

HK‐2 cells were seeded in 12‐well plates and cultured for 24 h. After incubation with SeCD, cells were digested with trypsin and centrifuged at 1000× g for 5 min. The supernatant was discarded and the cell pellet was resuspended in 100 µL PBS before excitation analysis by using the corresponding laser on a flow cytometer (CytoFLEX LX, Beckman, USA). A minimum of 10 000 cells were acquired from each sample across three independent experiments. Flow cytometry analysis was performed by using FlowJo v10.4.0. The relative mean fluorescence intensity (MFI) was calculated.

### Intracellular Fe^2+^ Determination

HK‐2 cells were placed in confocal dishes with glass bottoms and incubated for 24 h. After the indicated treatments as shown in each experiment, cells were stained with 1 µm FerroOrange working solution (F374, Dojindo) at 37 °C for 20 min in the dark. After being gently washed with PBS three times, cells were imaged by using a confocal microscope (N‐STORM, Nikon, Japan).

### DHE, MitoSOX, and MitoTracker Staining

HK‐2 cells were cultured in confocal dishes and incubated for 24 h. Then the cells were treated with 20 µm cisplatin for 24 h, 10 µm erastin for 24 h, or 1 µm RSL3 for 4 h, with or without 6 mg L^−1^ SeCD. Afterward, cells were incubated with 10 µm DHE working solution (S0063, Beyotime Biotechnology), 10 µm MitoSOX working solution (M36008, Thermo Fisher Scientific), or 250 nm MitoTracker working solution (M22425, Thermo Fisher Scientific) at 37 °C for 30 min in the dark. Subsequently, the working solution was discarded, and the cells were washed with PBS twice, and then visualized with a confocal microscope (N‐STORM, Nikon, Japan).

### Mitochondrial Membrane Potential Analysis by TMRE Staining

HK‐2 cells were cultured in confocal dishes for 24 h and treated with 20 µm cisplatin for 24 h, with or without 6 mg L^−1^ SeCD. The cells were gently washed with PBS, and then 200 nm TMRE working solution (T669, Thermo Fisher Scientific) was added. Then, the cells were incubated at 37 °C for 30 min and gently washed with PBS twice. HK‐2 cells were subsequently imaged by using a confocal microscope (N‐STORM, Nikon, Japan).

### DNA Extraction and Determination of mtDNA Copy Number

DNA was extracted from cultured cells by using a Universal Genomic DNA Kit (CW2298S, Cwbio). The copy number of mtDNA was quantified using quantitative real‐time PCR to evaluate the protection of SeCD on mitochondrial integrity under pro‐ferroptotic conditions. mtDNA was amplified with the following primers: Forward 5'‐ CAC CCA AGA ACA GGG TTT GT‐3' and Reverse 5'‐ TGG CCA TGG GTA TGT TGT TAA‐3'. The nuclear gene *Actin* was amplified as an internal reference with the following primers: Forward 5'‐ TAG AGG GAC AAG TGG CGT TC‐3' and Reverse 5'‐ CGC TGA GCC AGT CAG TGT‐3'. Quantitative real‐time PCR was performed in a total reaction volume of 10 µL containing 5 µL 2 × Universal SYBR Green Fast qPCR Mix, 2 µL ddH_2_O, 0.5 µL of each primer (10 µm), and 2 µL diluted DNA template. The reaction was performed at 95 °C for 3 min, followed by 40 cycles: 95 °C for 10 s, 60 °C for 20 s, 72 °C for 20 s.

### Bio‐TEM

HK‐2 cells were treated with 20 µM cisplatin for 24 h with or without 6 mg L^−1^ SeCD. The culture medium was discarded. Then, the cells were collected and fixed with pre‐cooled glutaraldehyde fixative (G1102, Servicebio) at 4 °C for 24 h. The cells were washed three times with 0.1 M phosphate buffer, and then fixed with 1% osmic acid (18456, Ted Pella Inc) for 2 h followed by dehydration in 30, 50, 70, 80, 90, 95, and 100% ethanol for 15 min each procedure. The samples were infiltrated sequentially in embedding medium (90529‐77‐4, SPI) and acetone (V/V = 1/1) for 1 h, embedding medium and acetone (V/V = 3/1) for 3 h, pure embedding medium overnight, and then polymerized at 60 °C for 24 h. Ultrathin sections were isolated, sequentially stained with uranyl acetate dihydrate (M019647, Micxy Reagent) solution and lead citrate (200964701, Sinopharm Group Chemical Reagent) solution for 10 min. The samples were visualized using a transmission electron microscope (HT7800, Japan) and images were captured.

### ATP Assay

HK‐2 cells were cultured in 6‐well plates, and treated with 20 µM cisplatin for 24 h with or without 6 mg L^−1^ SeCD. The culture medium was discarded, and HK‐2 cells were lysed with 200 µL lysis solution in an enhanced ATP assay kit (S0027, Beyotime Biotechnology). The cell lysate was centrifuged at 12 000× g for 5 min at 4 °C, and the supernatant was collected for subsequent ATP analysis. 20 µL supernatant was rapidly mixed with 100 µL working solution. ATP content was determined using a multifunctional enzyme labeler manufactured by PerkinElmer (EnSpire, America).

### Xenograft Tumor Model

To construct a xenograft tumor model, 1 × 10^7^ MCF‐7 cells were subcutaneously injected into BALB/c nude mice at the site between the fourth and fifth papillae on the lower left. The tumors were allowed to grow until they reached a volume of ≈130 mm^3^. Mice bearing xenograft tumors were then randomly divided into three experimental groups, and injected with 0.9% (w/v) NaCl solution, cisplatin, and cisplatin plus SeCD, respectively. Cisplatin was intraperitoneally injected twice a week at 4 mg kg^−1^ bodyweight, while SeCD was intravenously injected twice a week at 0.5 mg kg^−1^ bodyweight. The bodyweight and tumor volume were measured twice a week in the following 2 weeks post‐injection. The tumor volumes were calculated as V = ab^2^/2 (V, volume; a length of the tumor; b, the width of the tumor).

### Statistical Analysis

Data obtained from at least three independent experiments were presented as mean ± SD. Specific tests (one‐way ANOVA, two‐way ANOVA, or unpaired two‐tailed Student's *t*‐test) and the number of repeats are indicated in the figure legends. Differences were considered statistically significant when *p* < 0.05. The statistical analyses were performed with GraphPad Prism 8 software.

## Conflict of Interest

The authors declare no conflict of interest.

## Supporting information

Supporting Information

## Data Availability

The data that support the findings of this study are available from the corresponding author upon reasonable request.
