# Supplementary material for: Polyacrylic Acid‐Coated Selenium‐Doped Carbon Dots Inhibit Ferroptosis to Alleviate Chemotherapy‐Associated Acute Kidney Injury
Source: Adv Sci (Weinh). 2024 Apr 30;11(28):2400527. doi: 10.1002/advs.202400527 (PMC11267338; doi:10.1002/advs.202400527)
Supplement: Supplementary file 1 — Supporting Information [file ADVS-11-2400527-s001.pdf]

ADVANCED SCIENCE
Open Access

Supporting Information

for *Adv. Sci.*, DOI 10.1002/advs.202400527

Polyacrylic Acid-Coated Selenium-Doped Carbon Dots Inhibit Ferroptosis to Alleviate Chemotherapy-Associated Acute Kidney Injury

Jiahuan Li, Chengcheng Fu, Baoli Feng, Qingquan Liu, Jiangjiang Gu, Mohammad Nauman Khan, Lvhui Sun, Honghong Wu* and Hao Wu**

Supporting Information

Polyacrylic Acid-Coated Selenium-Doped Carbon Dots Inhibit Ferroptosis to Alleviate Chemotherapy-Associated Acute Kidney Injury

Jiahuan Li[1,2,#], *Chengcheng Fu*[2,3,4,5,#], *Baoli Feng*[1,2], *Qingquan Liu*[6], *Jiangjiang Gu*[7], *Mohammad Nauman Khan*[8], *Lvhui Sun*[1,2,*], *Honghong Wu*[2,3,4,5*], *Hao Wu*[1,2,*]

[1]State Key Laboratory of Agricultural Microbiology, College of Animal Science & Technology and College of Veterinary Medicine, Huazhong Agricultural University, Wuhan 430070, China

[2]Hubei Hongshan Laboratory, Wuhan 430070, China

[3]MOA Key Laboratory of Crop Ecophysiology and Farming System in the Middle Reaches of the Yangtze River, College of Plant Science & Technology, Huazhong Agricultural University, Wuhan 430070, China

[4]Shenzhen Institute of Nutrition and Health, Huazhong Agricultural University, Wuhan 430070, China

[5]Shenzhen Branch, Guangdong Laboratory for Lingnan Modern Agriculture, Genome Analysis Laboratory of the Ministry of Agriculture, Agricultural Genomics Institute at Shenzhen, Chinese Academy of Agricultural Sciences, Shenzhen 518120, China

[6]Department of Nephrology, Tongji Hospital, Tongji Medical College, Huazhong University of Science and Technology, Wuhan 430030, China

[7]College of Chemistry, Huazhong Agricultural University, Wuhan 430070, China

[8]School of Breeding and Multiplication (Sanya Institute of Breeding and Multiplication), Hainan University, Sanya, 572000, China

#These authors contribute equally

*Corresponding authors

E-mail address:

Lvhui Sun, lvhuisun@mail.hzau.edu.cn

Honghong Wu, honghong.wu@mail.hzau.edu.cn

Hao Wu, whao.1988@mail.hzau.edu.cn

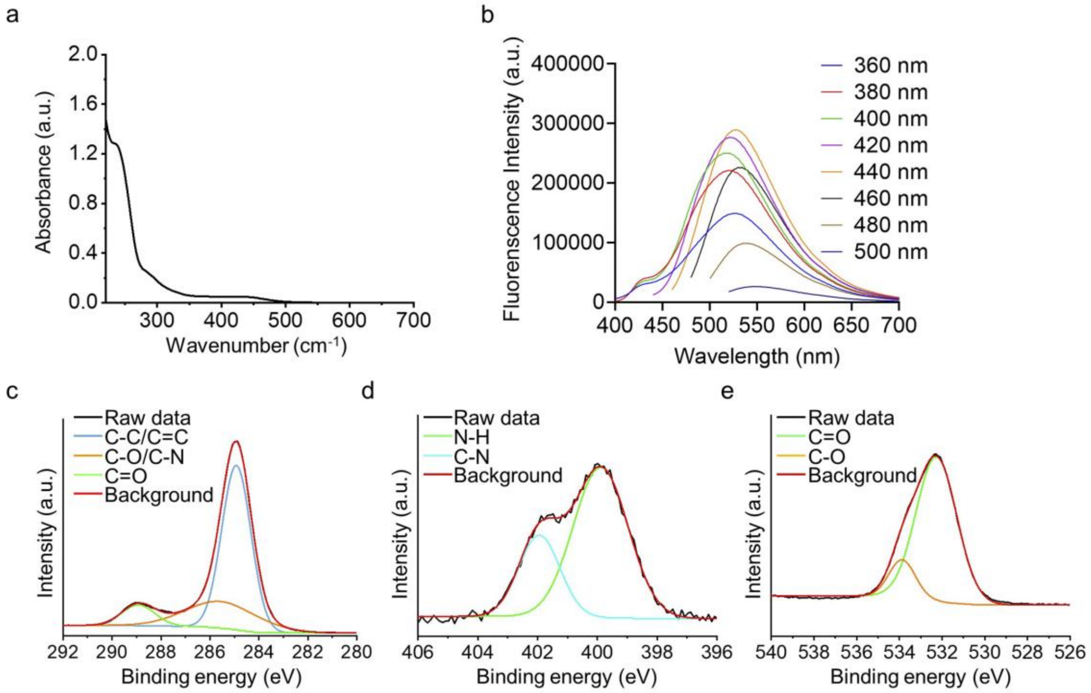

Figure S1. Characterization of SeCD. (a) UV/Vis absorption spectra of SeCD. (b) Fluorescence spectra of SeCD. (c-e) XPS spectrum of SeCD and high-resolution spectra of C1s (c), N1s (d), and O1s (e).

a

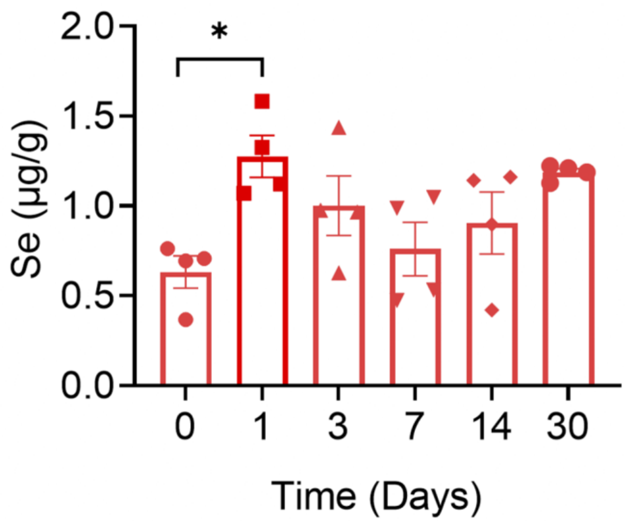

Figure S2. SeCD accumulates in the kidneys and is gradually cleared over time. (a) The selenium contents in the kidneys isolated from mice after 1, 3, 7, 14, and 30 days of single SeCD intravenous injection (0.5 mg/kg bodyweight) were determined by LC-AFS. All data are presented as mean ± SD (n = 4). Statistical significance was calculated using an unpaired two-tailed Student's t-test. * $P < 0.05$.

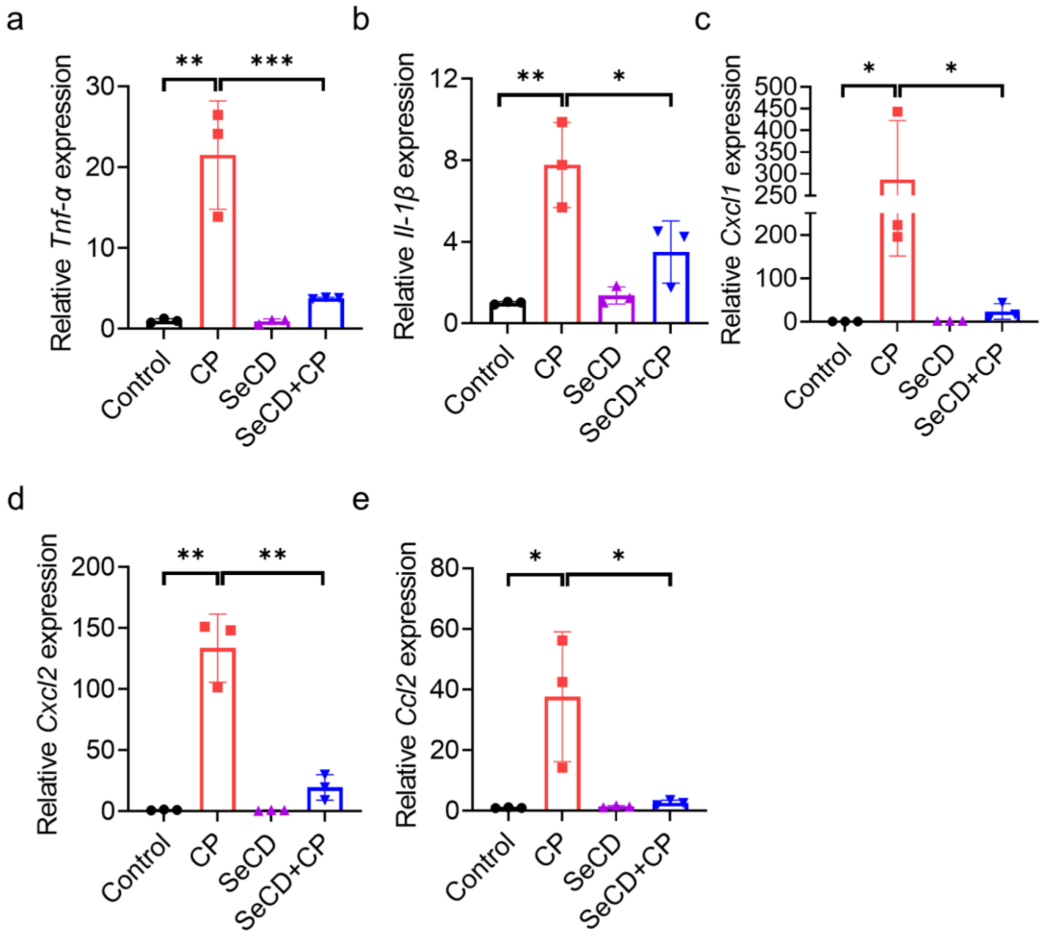

Figure S3. SeCD supplementation reduces the inflammatory response in AKI mice. (a-e) The relative mRNA expressions of *Tnf-α* (a), *Il-1β* (b), *Cxcl1* (c), *Cxcl2* (d), and *Ccl2* (e) in the kidneys isolated from each group of mice. All data are presented as mean ± SD (n = 3). Statistical significance was calculated using an unpaired two-tailed Student's t-test. * $P < 0.05$, ** $P < 0.01$, *** $P < 0.001$.

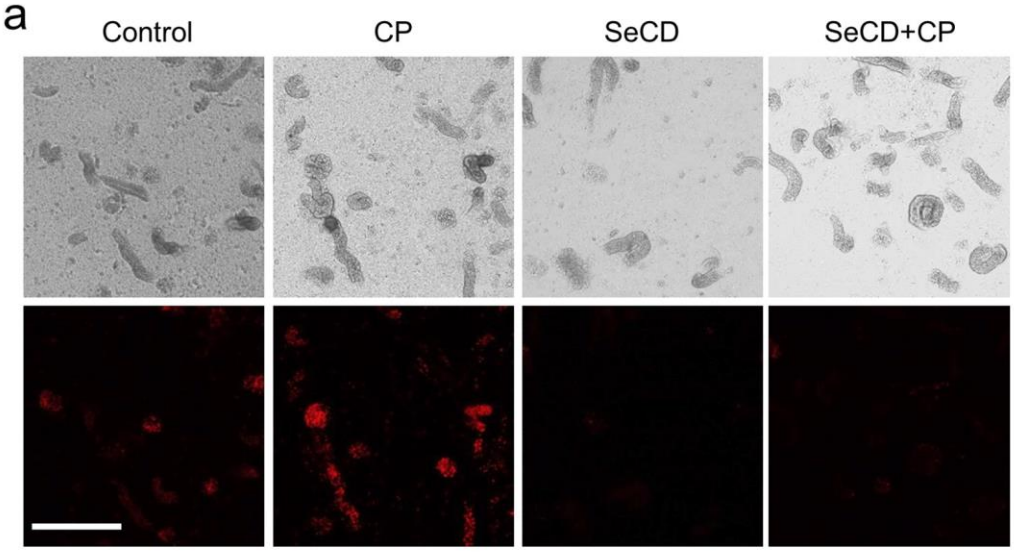

Figure S4. SeCD attenuates cell death in primary renal tubules. The primary renal tubules were treated with 20 μM CP with or without 6 mg/L SeCD for 24 h. PI staining was used to visualize cell death. Scale bar: 100 μm.

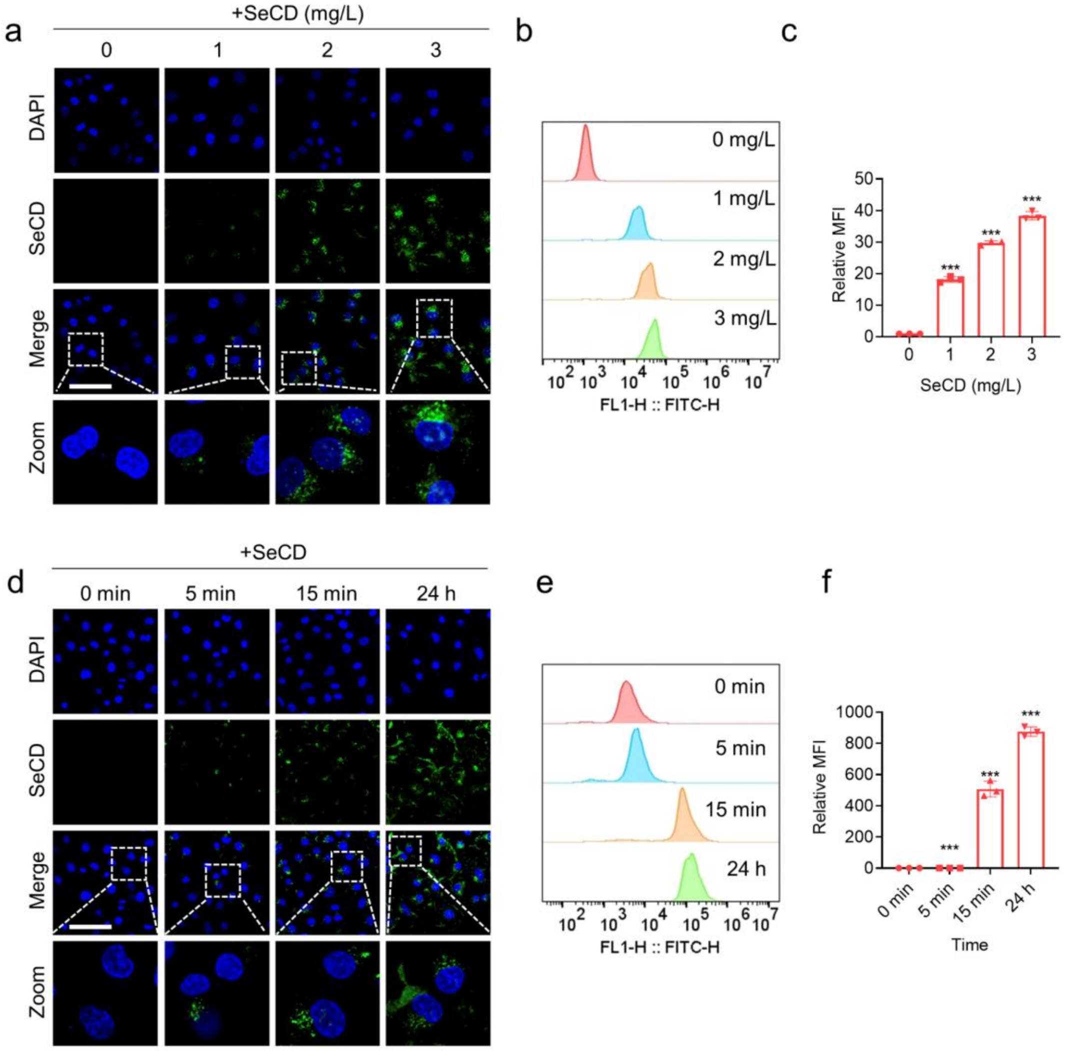

Figure S5. SeCD enters cultured cells rapidly. (a) HK-2 cells were incubated with the indicated doses (1, 2, and 3 mg/L) of SeCD for 4 h. The nuclei were labelled with DAPI. SeCD fluorescence is green. Confocal images showed SeCD uptake. Scale bar: 60 μm. (b, c) Cells were treated as in (a). Flow cytometry analysis was performed to show SeCD uptake (b), and the relative MFI was quantified (c). (d) HK-2 cells were incubated with 2 mg/L SeCD for the indicated time. The nuclei were labelled with DAPI. SeCD fluorescence is green. Confocal images showed SeCD uptake. Scale bar: 60 μm. (e, f) Cells were treated as in (d). Flow cytometry analysis was used to determine SeCD uptake (e), and the relative MFI was quantified (f). All data are expressed as mean ± SD (n = 3). Statistical significance was calculated using one-way ANOVA. *** $P < 0.001$.

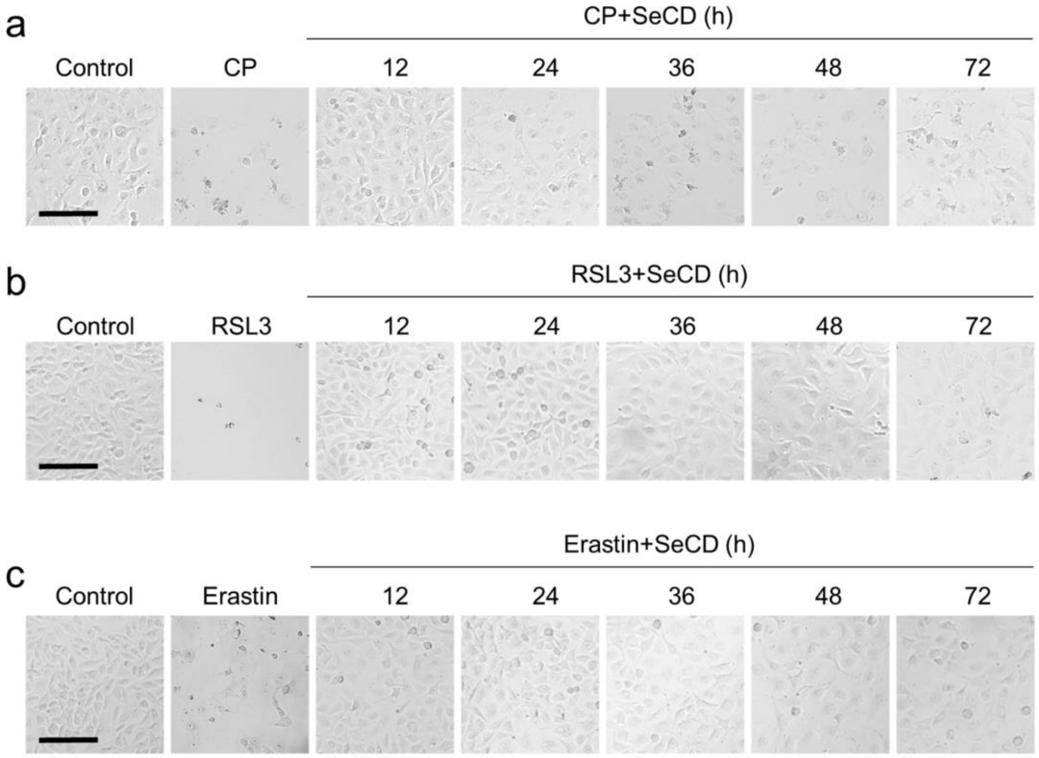

Figure S6. Long-term inhibition of ferroptosis by SeCD. (a-c) HK-2 cells were treated with 20 μM CP (a), 1 μM RSL3 (b), or 10 μM erastin (c) for the indicated time in the presence or absence of 6 mg/L SeCD. Microscopy imaging showed long-term protection of SeCD against ferroptosis. Scale bar: 100 μm.

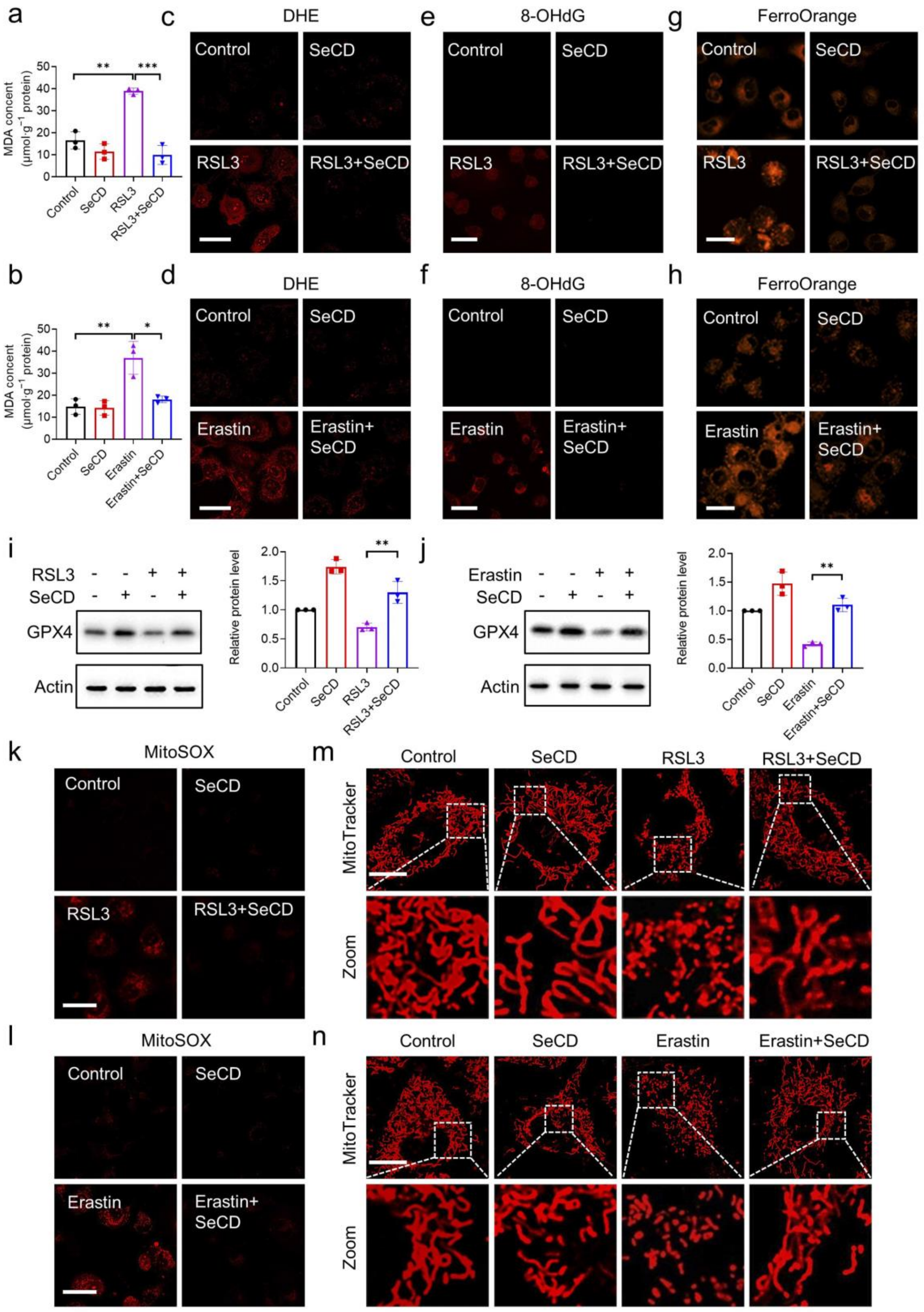

Figure S7. SeCD resists RSL3- and erastin-induced lipid peroxidation and preserves mitochondrial integrity. (a, b) HK-2 cells were treated with 1 μM RSL3 for 4 h (a) or 10 μM erastin for 24 h (b) in the presence or absence of 6 mg/L SeCD. The intracellular MDA levels were determined. (c, d) HK-2 cells were treated as in (a) and (b). Confocal images showed DHE staining in each group. Scale bar: 30 μm. (e, f) HK-2 cells were treated as in (a) and (b). The cells were fixed and stained with an anti-8-OHdG antibody. Representative confocal images were shown. Scale bar:

40 μm. (g, h) HK-2 cells were treated as in (a) and (b). Confocal images showed FerroOrange staining in each group. Scale bar: 30 μm. (i, j) HK-2 cells were treated as in (a) and (b). GPX4 expression was analyzed by Western blot. The relative protein levels were quantified and shown in the corresponding histogram. (k, l) HK-2 cells were treated as in (a) and (b), then stained with MitoSOX. Representative confocal images were shown. Scale bar: 40 μm. (m, n) HK-2 cells were treated as in (a) and (b), then stained with MitoTracker. Representative confocal images were shown. Scale bar: 15 μm. All data are presented as mean ± SD (n = 3). Statistical significance was calculated using an unpaired two-tailed Student's t-test. * $P < 0.05$, ** $P < 0.01$, *** $P < 0.001$.

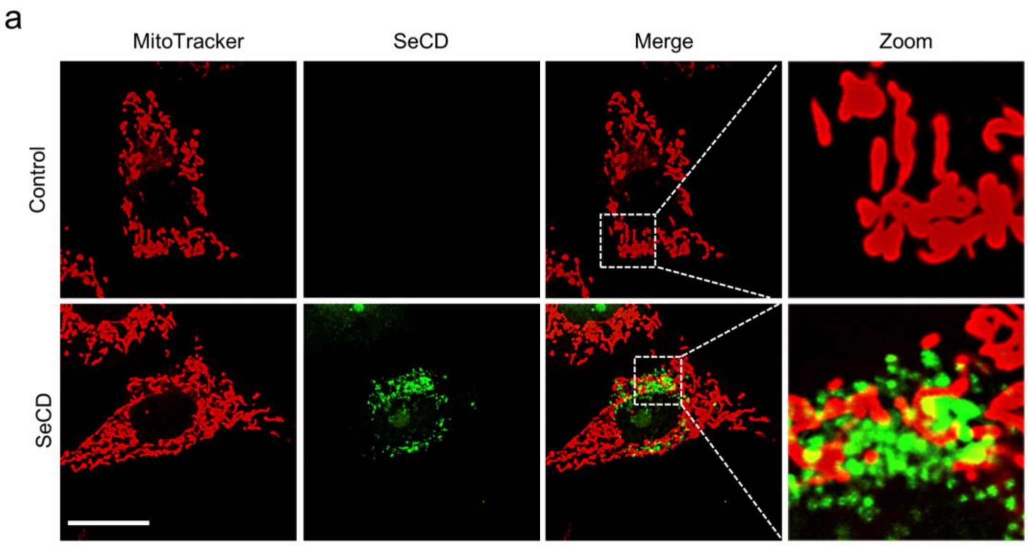

Figure S8. SeCD partially localizes in the mitochondria. (a) HK-2 cells were incubated with 6 mg/L SeCD (Green), then stained with MitoTracker (Red). Representative confocal images were shown. Scale bar: 20 μm.

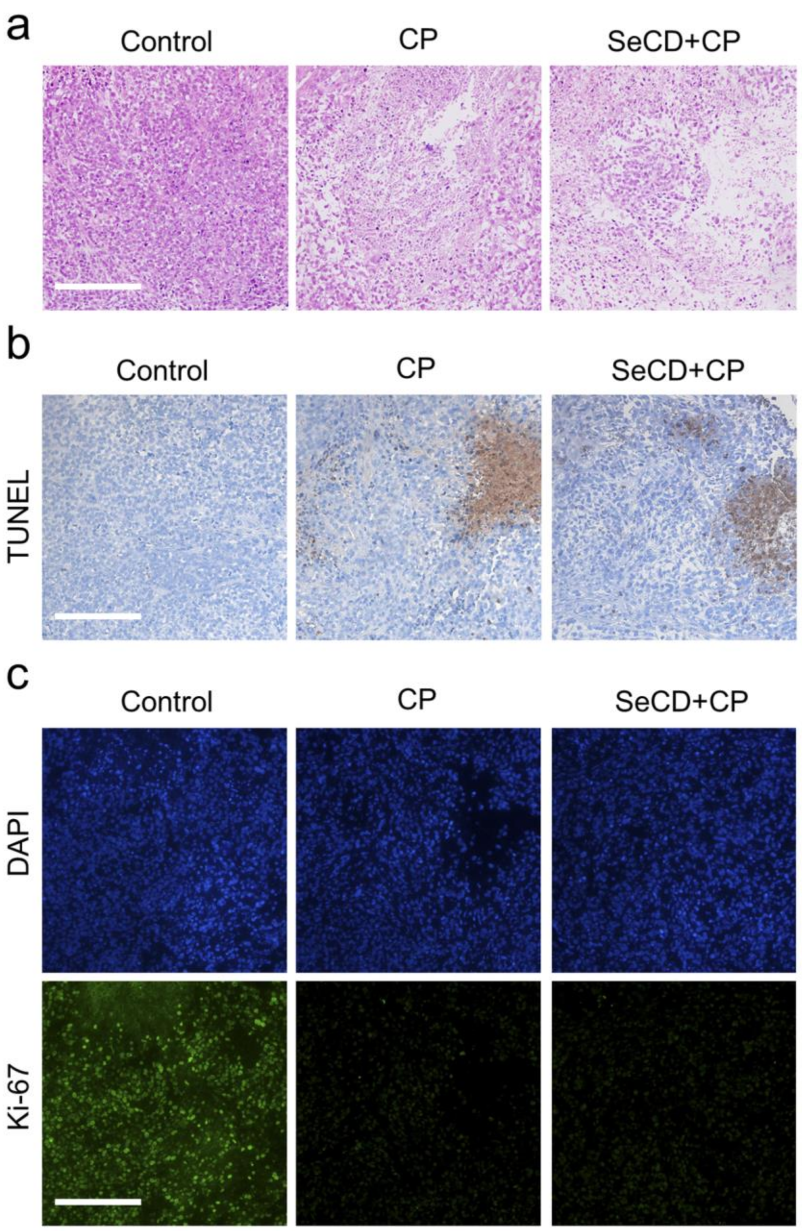

Figure S9. SeCD administration does not reduce the chemotherapeutic efficacy of cisplatin. (a) Representative H&E staining of tumors isolated from each group of mice. Scale bar: 200 μm. (b) Representative TUNEL staining in the tumors. Scale bar: 200 μm. (c) Immunofluorescence staining of Ki-67 in the tumors. The nuclei were stained with DAPI. Scale bar: 200 μm.

a

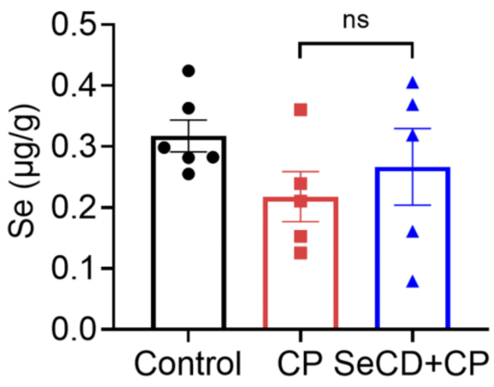

Figure S10. SeCD does not enrich in the xenograft tumors. The selenium contents in the xenograft tumors isolated from each group of mice were determined by LC-AFS. All data are presented as mean ± SD (n = 5-6). Statistical significance was calculated using an unpaired two-tailed Student's t-test. ns $P > 0.05$.

Materials:

Cisplatin (HY-17394) was purchased from MedChem Express. RSL3 (S8155), erastin (S7242), and Fer-1 (S7243) were purchased from Selleck. Tert-butyl hydroperoxide (TBH, 416665) was purchased from Sigma-Aldrich. Cell counting kit-8 (CCK-8, C0038), TUNEL apoptosis assay kit (C1098), and glutathione assay kit (S0052) were purchased from Beyotime Biotechnology. Krebs-Ringer liquid (PB180347) was purchased from Procell Life Science & Technology. H_2O_2 (FP17050002) was purchased from Sinopharm Group Chemical Reagent. Total RNA extraction reagent (Trizol, RK30129), ABScript III RT Master Mix for qPCR with gDNA Remover (RK20429), and 2 × Universal SYBR Green Fast qPCR Mix (RK21203) were purchased from ABclonal. ToloScript All-in-one RT EasyMix for qPCR (22107) was purchased from Tolo Biotech.

The primary antibodies used in this study are listed as follows: anti-GPX4 rabbit monoclonal antibody (ab125066) and anti-4-HNE rabbit polyclonal antibody (ab46545) were purchased from Abcam. anti-Actin rabbit monoclonal antibody (AC026) and anti-Ki-67 rabbit monoclonal antibody (A20018) were purchased from ABclonal. anti-8-OHdG (SC-66036) mouse monoclonal antibody was purchased from Santa Cruz Biotechnology. HRP-conjugated secondary anti-rabbit IgG (7074S) used in this study was purchased from Cell Signaling Technology. Fluorescent secondary antibodies used in this study include goat anti-mouse IgG Alexa Fluor™ 488 (A11001), goat anti-rabbit IgG Alexa Fluor™ 488 (A11008), and goat anti-mouse IgG Alexa Fluor™ 594 (A11012), which were purchased from Thermo Fisher Scientific.

Supplementary Table 1. Primer sequences used in this study.

Gene	Forward primer 5'-3'	Reverse primer 5'-3'
H-*Actin*	CACCATTGGCAATGAGCGGTTC	AGGTCTTTGCGGATGTCCACGT
H-*Gpx1*	CAGGAGAACGCCAAGAACGAAGAG	GCACCGTTCACCTCGCACTTC
H-*Gpx2*	CTTCTATGACCTCAGTGCCATCAGC	AGAGCGAAGCCACATTCTCAATCAG
H-*Gpx3*	AAAGAACTCCTGTCCTCCCACCTC	CCAGCGGATGTCGTGAACCTTC
H-*Gpx4*	CCGCTGTGGAAGTGGATGAAGATC	CTTGTCGATGAGGAACTGTGGAGAG
H-T*xrnd1*	CACCTGCGTGTCCTGTGCTTAC	CTGCCTGCCTTCTATTCACCAACTC
H-T*xrnd2*	CTTTGTTGACGAGCACACGGTTTG	CGCCCTCCAGTAGCAATGATGATG
H-T*xrnd3*	CATCATCATCGGTGGTGGTTCTGG	CCTGAGGTGACGGGACAACAAAG
H-S*elw*	TGATGTTCTCGTGGCTGCTGTTG	AGGGTGGGGTGGTGTGGATTC
H-S*elk*	CGCCTAAGTTCACGAGTTTGAGACC	CTGCCTCCGCCTCCTGAGTAG
M-*Actin*	CATTGCTGACAGGATGCAGAAGGA	TGCTGGAAGGTGGACAGTGAGGC
M-*Kim-1*	CAGGAAGACCCACGACTATTTC	GTGTGTAGATGTTGGAGGAGTG
M-*Ngal*	CCCTGTATGGAAGAACCAAGGA	CGGTGGGGACAGAGAAGATG
M-*Ptgs2*	GCGACATACTCAAGCAGGAGCA	AGTGGTAACCGCTCAGGTGTTG
M-*Tnf-α*	GGACTAGCCAGGAGGGAGAACAG	GCCAGTGAGTGAAAGGGACAGAAC
M-*Cxcl1*	GGCTGGGATTCACCTCAAGAACATC	TGAGTGTGGCTATGACTTCGGTTTG
M-*Cxcl2*	AACATCCAGAGCTTGAGTGTGACG	GGGCTTCAGGGTCAAGGCAAAC
M-*Ccl2*	CCACTCACCTGCTGCTACTCATTC	CTTCTTTGGGACACCTGCTGCTG
M-*1l-1β*	CACTACAGGCTCCGAGATGAACAAC	TGTCGTTGCTTGGTTCTCCTTGTAC
H-*Nd1*	TACTACAACCCTTCGCTGACG	AGCGATGGTGAGAGCTAAGGT
H-*Cox1*	TACGTTGTAGCCCACTTCCAC	GGGTGTAGCCTGAGAATAGGG
H-*Cytb*	CTAGCCAACCCCTTAAACACC	CAAGGACGCCTCCTAGTTTGT
